# Contemporaneous sample data tracking for the generation of genome edited cell lines

**DOI:** 10.1038/s41598-022-24928-6

**Published:** 2022-12-09

**Authors:** Anne L. Plant, Michael W. Halter, Jeffrey R. Stinson, Gretchen R. Greene

**Affiliations:** Materials Measurement Laboratory, National Institute of Standards and Technology, 100 Bureau Drive, Gaithersburg, MD 20899 USA

**Keywords:** Biological techniques, Biotechnology, Cell biology, Computational biology and bioinformatics, Stem cells

## Abstract

It is difficult to capture the large numbers of steps and details that often characterize research in the biomedical sciences. We present an approach that is based on commercial spreadsheet software so it is easily adaptable by the experimentalist. The approach is designed to be compatible with an experimentalist’s workflow and allows the capture in real time of detailed information associated, in this use case, with laboratory actions involved in the process of editing, enriching and isolating clonal gene-edited pluripotent stem cell (PSC) lines. Intuitive features and flexibility allow an experimentalist without extensive programming knowledge to modify spreadsheets in response to changes in protocols and to perform simple queries. The experimental details are collated in a table format from which they can be exported in open standard formats (e.g., Extensible Markup Language (XML) or Comma Separated Values (CSV) for ingestion into a data repository supporting interoperability with other applications. We demonstrate a sample- and file-naming convention that enables the automated creation of file directory folders with human readable semantic titles within a local file system. These operations facilitate the local organization of documentation and data for each cell line derived from each transfection in designated folder/file locations. This approach is generalizable to experimental applications beyond this use case.

## Introduction

The potential benefits of data sharing are great, and for the biological sciences there are many important activities focused on controlled vocabularies, standardized data formats, data repositories, etc., that play a critical supporting role (see^1^ for example). Key metadata are essential for interpreting the experimental data, but in biological research, often the collection of metadata is an afterthought to the design, execution, and extraction of data from the experiment. This situation exists, in part, because collecting detailed information about the many variables in a complex biological experiment, such as those involving cell lines, is highly challenging for the experimentalist especially when many steps are involved^2^.

Hand-written laboratory notebooks are still in common use in cell biology laboratories, and while many experimental details can be captured in this manner, text notes are not as conducive to exploring the relationships between variables and results as are electronic records that collect machine readable values. While electronic lab notebooks (ELN) can enable adequate digital record-keeping, they are cumbersome and expensive to implement because they often lack functionality and ease of updating that is desired by the experimentalist^3^. Recent efforts in publication of protocols such as help to provide a means of reporting experimental details by publishing protocols. While Nature Protocols https://www.nature.com/nprot/protocolexchange and bio-protocol https://bio-protocol.org/Default.aspx, for example, publish protocols as free-form text, protocols.io^4^ (https://www.protocols.io/welcome) helps to parse protocol text as variables. While these efforts are helpful, protocols do not provide records of actual activities in time, the details of which may change over the course of the study. As a result, the details of, and the relationships between processes, protocols and results can be lost, forgotten or misremembered. The loss of accurate information is a missed opportunity to gain benefit from the causal relationships in an experimental system to and to allow for the exploration of sources of irreproducibility through a database of collected information.

One of the most challenging areas of research is the use of cell lines. Cell lines may adapt to subtle differences in timing and details of handling and processing protocols, and inconsistencies in results from cultured cells may be the result of subtle differences that are not recognized as variables and therefore not recorded. In addition, unique cell lines are often created using genome editing which involves a number of complicated processes that can be aided by detailed protocol records. Genes may be “knocked-out” to assess the effect of their functional removal on control and development. Genes for fluorescent proteins or other proteins or peptides may be “knocked-in” to allow temporal observation of specific functions. Unintended events can occur in the process of developing genome edited cell lines that presumably result from some aspect of the editing process or culture conditions and passaging. There are many reports of different editing protocols that are designed to improve efficiency and accuracy of editing^5–9^, but it is difficult to assess these methods or to draw conclusions from them when protocol variables are not systematically examined, and data about editing and cell handling are not described in a systematic format that would be conducive to cross-study comparison. Because significant resources go into the development of culture methods and cell lines, thorough reporting that enables the discovery of the relationships between protocol variables and the final genomic and biological function outcomes would maximize the usefulness of these studies.

Here we detail a framework strategy using a spreadsheet program for conveniently designing a system for the collection of experimental protocol information that can serve as a collaborative analytical electronic lab notebook. Our example is designed to track and connect experimental and protocol metadata, data and samples that arise at intermediate steps during the development of clonal cell lines, but it is an approach that could be applied to other use cases that require keeping track of different actions at different times on multiple samples. We demonstrate this approach with a widely used commercial spreadsheet software package that makes it easy for experimentalists to prototype and refine the collection strategy and experimental details that are most appropriate to their specific experimental needs.

The philosophy of the approach presented here is twofold: to provides seamless integration of metadata collection into an experimental workflow, and to be sufficiently flexible so that the variables and other details can be entered and altered by an experimentalist who is not a computer programmer. Spreadsheet products incorporate many functions in an intuitive user interface making it easy to modify and optimize data collection and organization without database programming knowledge. At the same time, this approach can be made sufficiently robust to enable the collection of large amounts of experimental information over long periods of time, and the collected data can be easily imported into a more sophisticated database program for scaling to support more complex search and retrieval. We present this work as an example of a pragmatic approach that can be modified, customized, and built upon by experimental laboratories.

## Implementation

### Use-case driven development of the data model

For each experimental study that a lab embarks on, the goals and expected outcomes may be unique. There may also be ancillary observations that would be of interest to keep track of. However, not all details of an experiment are equally important, and burdening the user by trying to collect more information than necessary will risk fatigue and noncompliance. Therefor it is important to be thoughtful about how to select the protocol details to track. For our use case, we started by developing free text experimental protocols (structured processes) for this study, and these were used in part to help identify the discrete variables to be included in the spreadsheet fields as values. We also approached the challenge of which metadata variables to track by considering what analysis questions we might want to pose in the future. Our questions included how editing chemistries or procedures might influence unintended edits, and how cell handling might influence the efficiency of editing or the development of post-editing mutations during culture. Examples of the kind of queries one might want to pose about the effect of protocols on genomic edited cell lines are shown in Table 1. By formulating representative questions which explore the possible causes of experimental outcomes, the experimentalist is guided to collect the necessary variable data for eventual discovery through a systems-level search interface or by queries to a database. Discrete variables can be parsed and evaluated for relationships to one another and to non-discrete operational variables. As the field of editing pluripotent stem cells continues to mature, it may be possible to establish a community consensus list of queries and variables that would be widely applicable to a gene editing experiment, and which would enable comparison between different studies.

**Table 1:** Examples of questions for possible data queries to guide the collection of experimental variables.

How do different transfection protocols and handling of cells effect numbers and accuracy of edited clones?
What is the effect of different guide RNA sequences on numbers and accuracy of edited clones
Are rapidly dividing clones more often associated with chromosomal abnormalities?
Is passage number or feeding schedule associated with chromosomal abnormalities?
At what passage number do chromosome abnormalities tend to arise?
Are off-target or additional sequences at the insertion site associated with phenotypic abnormalities?
Can we perform the same protocol and receive the same efficiency and accuracy of editing?
Do different cell types show consistent differences in efficiency and accuracy of editing?

### Defining the experimental workflow

**Figure 1:**
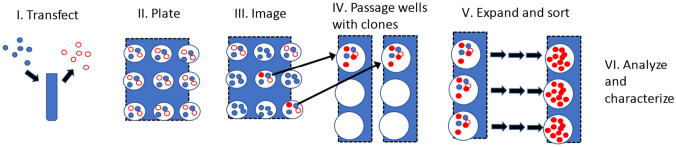
A schematic of the experimental process. In this use case, edited cells were selected based on expression of a fluorescent reporter, not on antibiotic resistance. Cells were transfected (I); examined for expression of edited fluorescent gene product (III); clones were expanded and enriched by iterative sorting with flow cytometry (IV-V); and analyzed (VI).

A schematic of our workflow with the steps for creating and purifying edited cell lines is shown in Fig. 1. The steps are: perform a transfection (I); distribute transfected cells into 96-well plates in the presence of un-transfected cells (II); image each well after 3 days to identify wells that contain one cluster of fluorescent cells indicating that they were edited (III); passage and expand the cells from those wells (IV); flow sort them to enrich for fluorescent cells, and iterate expansion and sorting processes until populations exhibit a single highly fluorescent peak by flow cytometry (V). Purified cell lines were then expanded to create a bank of cells, and samples were taken for analysis of genomic modifications and purity. Cell populations were also frozen and stored at intermediate steps to enable characterization of intermediate populations and in the event that lines needed to be recovered due to an unexpected loss of the cell line during processing.

### System architecture design

Our workflow and data requirements presented several key challenges. We wanted to capture detailed information about the genome engineering process and record the actions performed on each transfection and resulting clones over time through iterative steps of expansion and enrichment, freezing and genomic analysis. In addition, we wanted to be able to link data associated with each cell population with the metadata describing its process history.

**Figure 2:**
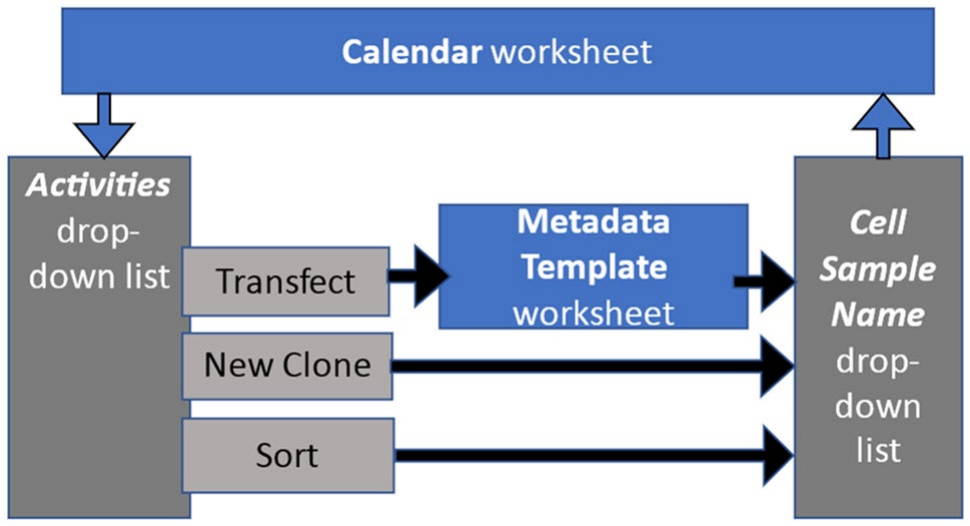
Components of the user interface program for collecting metadata. The user can enter data on two interrelated worksheets, the transfection **Metadata Template** worksheet and the **Calendar** worksheet (shown in dark blue). Cell sample names are automatically updated according to the naming convention in response to certain selected activities, and this list updates the ***Cell Sample Name*** drop-down list on the **Calendar** for selection by the user.

We employed the widely used commercial spreadsheet software product, Microsoft Excel to create our user interface and to provide low-level database functions. [The use of trade and product names is not intended to imply recommendation or endorsement by NIST, nor is it intended to imply that the materials, software, services or equipment are necessarily the best available for the purpose.] The real-time collection of protocol metadata in this study occurred within a workbook that consists of several kinds of interrelated worksheets (see Fig. 2).

Preliminary experiments helped to establish an optimum workflow for the experimentalist, which provided the basis for a user interface format. In our case, the daily collection of information about activities to be performed involved making entries on a calendar, which served as an organizational tool for the day’s work. Therefore, we chose a calendar template with which to prototype data input. Activities to be performed with cell samples under study were entered into the calendar for that day either at the beginning of the day, or at any time. Once we were satisfied with the basic processes for data entry, we engaged a commercial programming company (Excel and Access, LLC, Fullerton, CA) to formalize our prototype to a fully functional experimental workflow software program employing Visual Basic Application (VBA) coding to improve navigation and make the functionality robust and efficient.

The Excel program file, TransfectionTracker1221.xlsm, with its supporting VBA code and a README document, are publicly available at https://github.com/usnistgov/TransfectionTracker. The version of Excel used is Microsoft Excel for Microsoft 365 MSO (16.0.13127.21490) 32-bit. The README document (which is also provided as note S1) contains detailed instructions for how to use the program and many of the features that are referred to in this manuscript. [In this document, **Bold** lettering indicates the name of a worksheet; ***Bold Italics*** indicates an input available on a worksheet such as ***Activity*** and ***Cell Sample Name*** on the **Calendar** or ***Data*** from the toolbar at the top of the page; *Italics* indicates a choice of input often from a drop-down list; Underline indicates examples of free text.] The Excel program file consists of a workbook of worksheets that contain data from three replicate transfection experiments. Descriptions of all worksheets in the workbook are provided in Table 2 and in the **Documentation** worksheet in the Excel workbook. The user can add data to this workbook by entering activities into the **Calendar** worksheet and can update other pages as described in the README document. An additional Excel file, named TransfectionTracker1221.clean.xlsm, which contains no data, is also provided so the user can create a new body of data and modify templates if desired to create a bespoke system for their specific laboratory activities.

**Table 2:** Worksheets in TransfectionTracker1221.xlsm and their function.

Worksheet name	Worksheet type	Description
Documentation	Documentation	Key commands and workflows
Calendar	Data entry	Enter daily activities
Metadata Template	Template	Blank template for entering transfection details
20200113mChOCT4sg2	Completed template	Example metadata for a specific transfection. A unique metadata worksheet is created for each transfection
20200120mCHOCT4sg2	Completed template	Example metadata for a specific transfection. A unique metadata worksheet is created for each transfection
20200220mChOCT4sg2	Completed template	Example metadata for a specific transfection. A unique metadata worksheet is created for each transfection
Data	Report	Tabular form of all entries on **Calendar**
ActivityList	List of terms	Activities that can be selected on the **Calendar**
Cell Samples	Report	All samples created and that can be selected on the **Calendar**
Data validation criteria	List of terms	Source of all drop-down lists on the **Metadata Template**
TransfectionsReport	Report	Simple query of all records in **Data** associated with activity *Transfect*
FreezeReport	Report	Simple query of all records in **Data** associated with activity *Freeze*
DiscontinueReport	Report	Simple query of all records in **Data** associated with activity *Discontinue*
ExtractDNAReport	Report	Simple query of all records in **Data** associated with activity *ExtractDNA*
20200113mChOCT4sg2Report	Report	Records from **Data** associated with selected ***Cell Sample Name***
20200120mCHOCT4sg2Report	Report	Records from **Data** associated with selected ***Cell Sample Name***
20200220mCHOCT4sg2Report	Report	Records from **Data** associated with selected ***Cell Sample Name***
Passage#s200113_C6	Report	Activities from **CloneReport** selected to allow counting of numbers of passages of that cell line
Passage#s200120_B9	Report	Activities from **CloneReport** selected to allow counting of numbers of passages of that cell line
Passage#s2020220_A7	Report	Activities from **CloneReport** selected to allow counting of numbers of passages of that cell line
Passage#s200220_C6	Report	Activities from **CloneReport** selected to allow counting of numbers of passages of that cell line
Passage#s200220_C10	Report	Activities from **CloneReport** selected to allow counting of numbers of passages of that cell line
AllClonesIist	Report	List of all unique cell samples
ClonesSummaries	Report	Compiled data for all clones
Notes	Report	Daily notes recorded on **Calendar** (contains no data)

**Figure 3:**
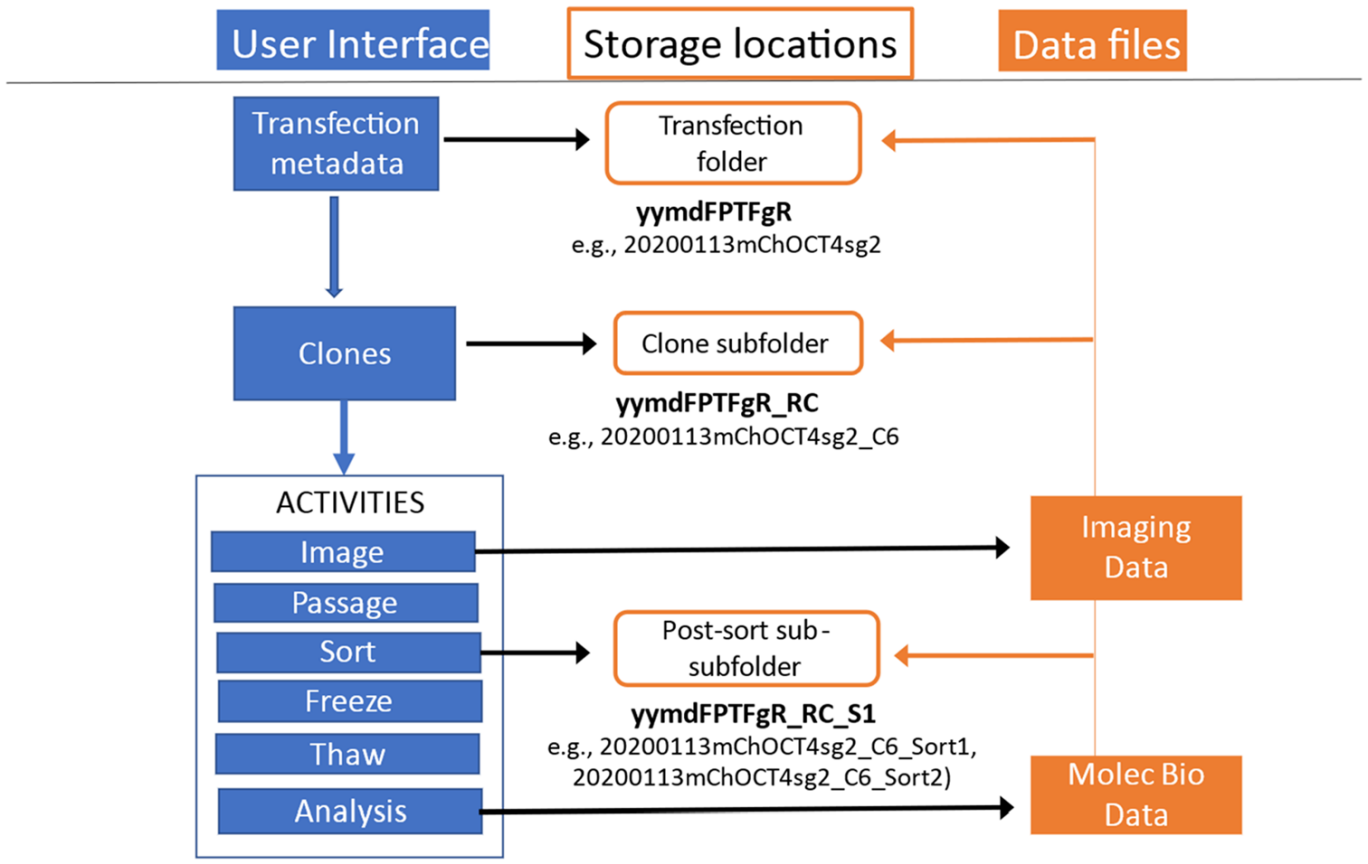
Schematic of the data model and naming convention. Various activities were performed on each cell sample. Initiating a transfection resulting in generating a unique transfection designator, or cell sample name, which served as the base name for subsequently derived cell samples. Updated cell sample names were created by appending terms to the transfection designator to indicate activities performed on the sample. Using a cell sample-naming convention, storage location folders were automatically generated with those unique sample designators, providing a local persistent identifier and an organizational structure for storing related sample-specific data. In this naming convention, FP represents fluorescent protein, TF represents transcription factor, and gR represents guide RNA. Row “R” and column “C” locations in 96-well plates where edited cells were observed (e.g. “_C6”) provided an appended designator for that cell sample that was isolated and further acted on. Flow cytometry sorting was used to enrich and expand populations of edited cells, which were indicated by appending to the sample name the designation “_ Sort#”. The “#” was incremented automatically for samples as they were subjected to subsequent sorting events.

Each transfection performed can result in multiple cell lines. To facilitate organizing data for different cell samples that result from a transfection, several lines within the VBA code can be activated to automatically create folders and subfolders on a designated network drive; this is described in the **Documentation** worksheet. Nested folder names are based on a naming convention (described below and in Fig. 3) that provides a unique but related name to each derived cell sample. This method for automatically naming nested folders and assigning a specific data storage location that serves as a local persistent identifier facilitated keeping all data for each related sample unambiguously organized.

While the current example shown is demonstrated with a Windows operating system, Excel is readily available for Mac based systems. The auto generated directory path can be adapted for Linux OS filesystem syntax by adding a configure option for the worksheet entry points.

There is often a trade-off between sophistication and flexibility in software. The built-in functions of this spreadsheet program allow a non-programmer to design a bespoke data intake process, provide access to sophisticated data entry and manipulation features, and allow a level of flexibility that makes it possible for the average user to evolve the data intake process. At the same time, the program is sufficiently powerful that a knowledgeable programmer can add sophisticated navigation and operation features.

### Data model and file-naming convention

Establishing connections between the metadata describing the activities performed, and the resulting imaging and characterization data, required the creation of unique identifiers for transfection occurrences, edited clones and cell subpopulations. A schematic of the connection between the user interface for metadata collection and the naming scheme for folder locations for data storage is shown in Fig. 3. User-initiated data input into a spreadsheet results in automatic updating of sample names and creation of directory folders, allowing association of data for each cell population with the experimental and protocol metadata that described its handling.

The naming convention creates human-readable filenames which include key data about the sample, such as the date of transfection, the fluorescent protein, the gene of interest, the guide RNA, and process steps such as clone identification and cell sorting (as shown in Fig. 3). Specifically, each transfection is designated with a name that is a concatenation of the year (in four digits), the month (in 2 digits), and the day (in 2 digits) corresponding to the date of the transfection. This is followed by an abbreviation for the fluorescent protein used in the construct, i.e., mCh (for mCherry). This is followed by an indicator for the gene that was being modified (OCT4), and an indicator of the guide RNA that was used (sg2). These details are collected on the Metadata Template with the aid of drop-down lists, which are controlled through the **Data validation criteria** worksheet (which is discussed in detail below). As transfected cells were found by fluorescence imaging, they were identified with their position in a 96-well plate; thus transfection designators were appended with “_C6” or “_A2” for example, and these appended names followed these samples through subsequent processing steps. Our workflow included steps for enriching transfected cells by flow sorting. Cell sample names were appended to indicate that the sample had been sorted, and how many times, i.e., “_Sort1” or “_Sort4”, where the number of the sort was automatically updated when the ***Activity**** Sort* was selected to be performed on a previously sorted cell sample. This naming scheme provides sufficient information to allow identification of the sample through its name, to unambiguously track the activities and timelines for the sample, and to be able to verify through the records exactly where the sample was in the development process when it was stored or tested or analyzed. Having a naming convention such as this enabled simple data queries and parsing of data through the spreadsheet program via filtering and organizing data based on activities and/or files name components. This naming convention can be customized by incorporating bespoke human readable characters that are specific to a particular laboratory situation or experiment, enabling a researcher to identify samples and reduce ambiguity.

The naming convention enables the organization of all information about a cell sample at every stage of development of a cell line in one place on a network drive. This is accomplished by assigning a network address as described in the **Documentation** worksheet and automating the creation of folders and subfolders in that location through the VBA code in Excel as new cell samples are created. Thus, when a new transfection metadata worksheet is created, a new folder can be created on the designated network drive named with the resulting new cell sample name. When positive transfectants are identified by fluorescence microscopy and designated as clones, a subfolder within the original folder can be created with the new cell sample name (i.e., the transfection designator appended by the well location of the fluorescent clone). Sub-subfolders were created each time a clone-containing cell sample was sorted, and these were named with the updated cell sample name that indicated sort number. Data pertaining to the cell sample at different stages of its development, including image data, flow cytometry data, and genomic analysis data, could be placed in the appropriate folder. In our use case, some data such as image data were written directly to the appropriate folder from the instrument; other data, such as the results from genomic analysis which was performed offsite needed to be manually copied to the appropriate network folder. Having a method for automatically assigning a specific data storage location facilitated having a place to keep all data for each sample unambiguously related.

## Results and discussion

Our approach to the collection of metadata was driven by the following principles: convenience for the experimentalist and seamless integration into the experiment workflow; benefit to the experimentalist by reducing the need for making notes on paper or other media and providing automatic performance of common calculations; reducing errors and fatigue in data entry by providing controlled lists from which the experimentalist can select variable values; and linking sample metadata in the spreadsheets with experimental data in a directory structure through an automated naming convention. For this kind of study, it is also important that the experimentalist has a place for descriptive notes in an unstructured text format for information that is not easily captured as machine-readable variables.

### Data entry worksheets

**Figure 4:**
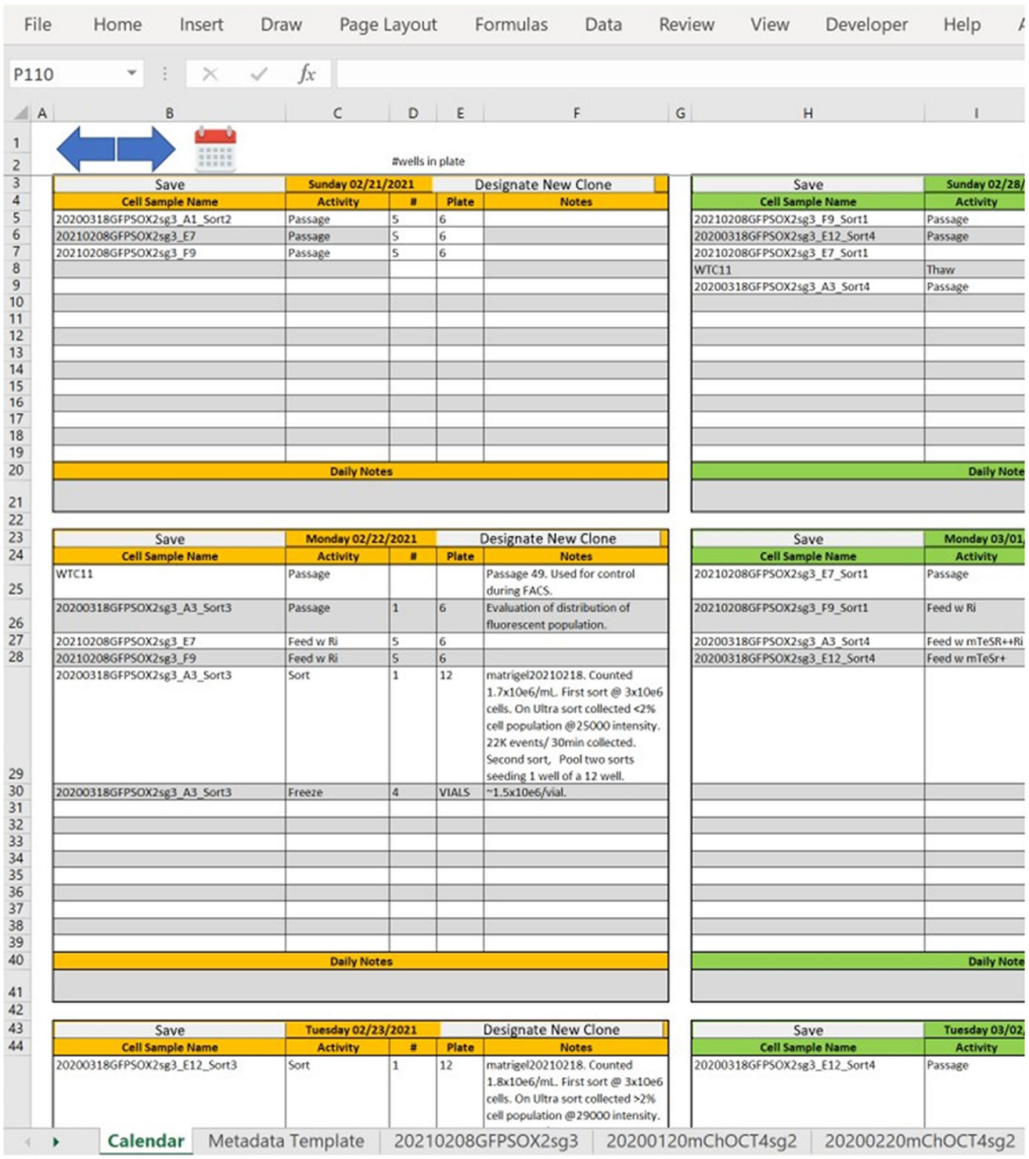
The Calendar worksheet. This is a partial view of the worksheet for capturing daily experimental and maintenance activities. The user can navigate to any date, choose entries from drop-down lists, and add free text notes.

**Figure 5:**
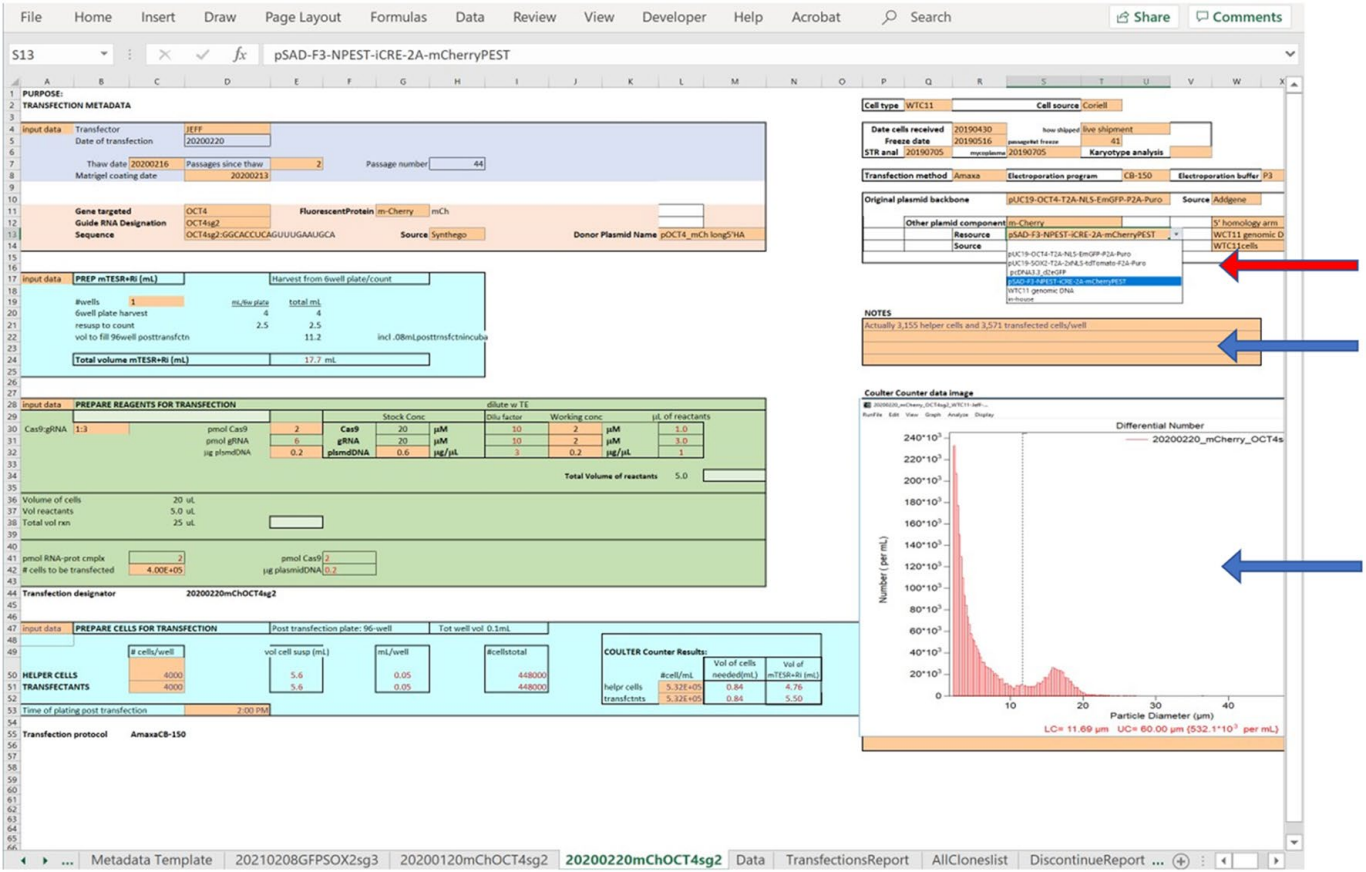
The Transfection Metadata Template worksheet. This is a partial view of the worksheet showing a drop-down menu, indicated by the red arrow, of plasmid component sequences to choose from, and the areas of the worksheet that provide space for free text and for insertion of an image (e.g. of data from a cell counter), indicated by the blue arrows. The green box contains automated calculations of amounts and volumes of materials to use in the transfection in response to user inputs. Limits are imposed on some of the values to conform with experimental protocol. Cells colored in orange are cells where the user inputs data by choosing from a drop-down list or by entering values or free text.

The experimentalist enters values for variables into two different interconnected worksheets as shown in Fig. 2, one that is templated as a calendar (the **Calendar** worksheet, shown in Fig. 4) and is used to capture daily activities performed on cell populations that result from a transfection, and another for input of transfection variables (the **Metadata Template** worksheet, shown in Fig. 5).

From the **Calendar** worksheet, when the user chooses the ***Activity**** Transfect*, a **Metadata Template** is presented and data corresponding to the transfection are entered by the user. Every transfection results in a new worksheet. As specific data for a transfection are entered into the **Metadata Template**, a unique designator for that transfection is created by concatenation of key data entered on the worksheet according to the established naming convention, and the resulting transfection metadata worksheet tab is updated with the unique transfection designator. This new sample name automatically populates another worksheet, the **Cell Samples** list. For each subsequent new transfection, a new transfection metadata worksheet is created, resulting in another tab named with the new transfection designator, and the addition of a new cell sample name to the **Cell Samples** worksheet. The updated **Cell Samples** worksheet list populates the drop-down list of ***Cell Sample Name*** on the **Calendar** worksheet. Figure S1 shows a view of the **Cell Samples** worksheet containing cell sample names, and a view of the **Calendar** worksheet showing the drop-down list of cell sample names that can be chosen. If a new transfection is initiated that is similar to a previous one, the user can select an existing transfection metadata worksheet to serve as a template. Only new information needs to be entered into the new transfection metadata worksheet, and the result is an additional and uniquely named transfection metadata worksheet tab. Previously transfected cell populations can be selected from the ***Cell Sample Name*** drop-down list on the **Calendar** worksheet as activities are performed on them; some of those activities (***Designate a New Clone***, and *Sort*) result in appending the cell sample name to indicate the activity and assign a new cell sample; the new cell sample name is automatically added to the drop-down list. Thus, the drop-down list for ***Cell Sample Name*** includes all unique cell samples derived from every transfection. The most recently named cell sample appears at the top of the drop-down list.

The **Calendar** worksheet (Fig. 4) guides the entry of information about daily activities carried out with each cell population and serves as a planning and organizational tool for the experimentalist. The user can choose any day, current, past, or future in which to make entries. The **Metadata Template** (Fig. 5) captures information about the editing reagents and the transfection protocol and provides space for unstructured notes and images. In addition, functionality was embedded in the **Metadata Template** to provide some calculations that guide the experimentalist in setting up the transfection reaction conditions.

We found that interacting with the **Calendar** and **Metadata Template** worksheets required no more time or effort than entering written information into a notebook. Many features of the program simplified the recording of data and the organizing of the workflow including the use of drop-down lists for selecting options, the use of previously filled **Metadata Template** worksheets for similar transfection experiments, the inclusion of automated calculations in the **Metadata Template** worksheet, and the automated compilation of information from multiple worksheets in the form of report worksheets using built-in software functions.

Both the **Calendar** worksheet and the **Metadata Template** (including the metadata worksheets created for specific transfections) have space for the experimentalist to add free form text, so all information about the activities can be captured even if there is not a specific worksheet cell for it. In that way, all information deemed important by the experimentalist can be included in the worksheet for that experiment. In addition, data and information in the form of images, graphs, and screenshots from instruments or other sources, can be pasted into the **Metadata Template** worksheet. Free form text also can be saved as ***Notes*** on the **Calendar** worksheet and these notes then appear on the **Data** worksheet associated with the **Calendar** entry.

### Restricted data entry

Both the **Calendar** worksheet and the transfectio*n* **Metadata Template** worksheet make use of drop-down lists which control and facilitate data input by limiting the values the experimentalist can select. The drop-down lists are pre-configured and reside in other worksheets. The purpose of the drop-down lists is to prevent the entry of unintended and erroneous values into the worksheet and to maintain consistent vocabulary. Drop-down lists can also simplify the experimentalist’s job of data entry. For example, the activities that can be chosen on the **Calendar** are limited to the entries on the **ActivityList** worksheet which are shown in Table 3.

**Table 3:** The activities that can be selected for any cell sample on any day from the drop-down list in the **Activity** column in the **Calendar** sheet.

Activity
Transfect
Feed w Ri
Image
Passage
Sort
Freeze
Thaw
Extract DNA
Feed w mTeSR++Ri
Feed w mTeSr+
Discontinue
Send out for analysis

Drop-down lists can be modified in a controlled manner in several different ways. The drop-down list for ***Activity*** on the **Calendar** worksheet can be altered from the **ActivityList** worksheet simply by removing or adding activities to the existing list; the new activities will show up in the drop-down list in the **Calendar**.

The **Cell Samples** worksheet, which provides a drop-down list from which to choose the ***Cell Sample Name*** on the **Calendar** worksheet as described above, is modified and controlled through the program in response to user input as described in detail in “Data entry worksheets” section above. When cell samples are acted on in ways that modify them, their names are appended with information about the activity *Transfect* or *Sort*, or when the ***Designate New Clone*** button is activated in the **Calendar** worksheet. The continuously updated list of cell sample names is available in the **Cell Samples** worksheet and appears as a drop-down list in the **Calendar** worksheet (Fig. S1).

The entries for drop-down lists for the **Metadata Template** worksheet are contained in the **Data validation criteria** worksheet (Fig. S2). Figure S2 shows some of the lists of terms from which the experimentalist selects entries. We designed the **Data validation criteria** worksheet to contain a number of variables that we anticipated would be important to record, and for each variable we developed a list of likely choices. Transfection variables such as which guide RNA was used, or which electroporation program was used could be controlled by highlighting the appropriate cell on the **Metadata Template** and choosing from the toolbar *Data/Data tools/Data Validation* to select the list of options to appear in a drop-down list for that cell. Additions can be made to those lists when necessary for inclusion in the drop-down lists in the **Metadata Template** worksheet in a controlled fashion using the *Protect* and *Unprotect* functions.

Worksheets and cells within worksheets can be protected with a password to prevent unintended changes, and unprotected to allow intentional changes to be made. Protection can be managed from the Excel toolbar at the top of the page by choosing the toolbar tab *Review/Protect Sheet*. One can select which cells in a worksheet are unlocked, allowing the user to input text or values into those cells, select inputs from drop-down lists, or add unstructured text or images. In the example **Metadata Template** worksheet, the unlocked cells are colored orange as shown in Fig. 5. Indicated with blue arrows in Fig. 5 are input fields for free text notes and for pasting images of data, for example from a cell counter or flow cytometer. Some cells in the worksheet that are locked contain the results of calculations using numbers that are entered into other cells, allowing, for example, the calculation of solution volumes based on input values for concentrations, and concentrations of stock solutions required to achieve appropriate reaction volumes. The entries in these cells are in red font. Other locked cells contain formulas or logic tests that are used to provide warnings if, for example, the volumes of reactants are greater than can be accommodated in the transfection vessel. Other cells are locked for the convenience of the experimentalist because they contain values that are important variables associated with the protocol but aren’t expected to change frequently. Controlling the locked or unlocked state of individual worksheets cells is achieved by unprotecting the workbook with a password, and highlighting the cells and accessing the *Protection* tab in the *Format Cells* function with a right mouse click. The worksheet can then be protected again to avoid unintentional changes.

More details about controlling data entry are provided in README at https://github.com/usnistgov/TransfectionTracker.

### Worksheets for organizing and comparing data.

Data for each transfection are saved on separate worksheets. All cell processing data for all transfections are compiled on the **Data** worksheet. Additional worksheets can be created to organize and report compiled data with filter, sorting, and query tools from the toolbar. Worksheets can be hidden using the *Hide* and *Unhide* function accessed with a right-click on the worksheet tab. This feature hides worksheets from view, allowing the user to focus on the current worksheets while still being able to easily access the data by unhiding worksheets. The **Data** worksheet (Fig. S3) organizes all data collected for all samples and activities entered in the **Calendar** worksheet in a table format. This feature facilitates converting the spreadsheet data into XML, JSON or CSV format for query in a dedicated database. It contains dates, cell sample names, activities, numbers of wells and well-plate size when appropriate, notes, and indication of the user and the user computer address as provided by the operating system if desired (see the **Documentation** worksheet in the TransfectionTracker1221.xlsm Excel file at https://github.com/usnistgov/TransfectionTracker for details of how to activate this feature).

Queries can be easily performed, and reports can be generated, by selecting the entries in the **Data** worksheet and choosing the toolbar function ***Data***. A Query Editor dialog box appears and simple queries based on a selection from the ***Activity*** column can return the appropriate records on a new worksheets such as **TransfectionsReport**, **FreezeReport**, **DiscontinueReport** as shown in the example workbook, TransfectionTracker1221.xlsm, at https://github.com/usnistgov/TransfectionTracker. These worksheets contain all entries for all cell samples that have undergone these activities. By selecting from the ***Cell Sample Name*** column, worksheets can be generated that include all entries for all cell samples resulting from a particular transfection, as in the **20200113mChOCT4sg2Report** worksheet. These worksheets provide a list of all activities performed over time on cell samples derived from a particular transfection. These report worksheets can be updated as new data are added to the **Calendar** (and therefor to the **Data** worksheet) using the toolbar option ***Data***/*Refresh*. Two of these worksheets are shown in Fig. S4.

Alternatively, the data can also be organized by copying and pasting them into a new worksheet and then sorting and filtering the entries from the toolbar alphabetically, by date, or by other contents of the columns. A useful manipulation is to calculate the numbers of passages a cell sample has undergone. Five worksheets with names beginning with **Passage#s** are in the example workbook, each containing data from a different clone from one of the 3 transfections. From the **…Report** worksheet for each transfection, the rows of data that follow the ***Activity**** Transfect* for a particular transfection date were copied into the worksheet. The data rows were then filtered based on entries in the ***Activity*** column that were relevant to passaging, namely *Passage*, *Freeze*, and *Thaw*. These entries provided a count of how many passages had occurred for this sample when it was frozen, thawed, or sent out for analysis. An example of this is shown in Table S1. The passage number of a frozen bank and of samples used for genomic analysis is unambiguous as a result, or if ambiguities are discovered, they can be tracked down or noted. The numbers of passages that different cell lines have undergone can be easily compared to one another.

A worksheet entitled **ClonesSummaries** is part of the example workbook and the contents are presented as Table S2. This worksheet collates many of the results associated with the clones created. Some of the data in the table were added through queries, other data were manually entered. The worksheet allows identification of which clones were analyzed and which were discontinued, the date that genomic analysis was initiated and the passage number of the cell sample at that time, and results such as copy number of the fluorescent protein sequence, copy number variants, results of PCR analysis in the insertion site, qualitative appearance of the cell line, and other analyses such as for mycoplasma and for STR markers. By adding the URL where the data for each clone is saved, it is easy to identify and access the primary data for each clone unambiguously. As the worksheet table shows, of 15 clones that were isolated, 5 were successfully carried through enrichment and expansion. Two of those were confirmed to have a large number of extra bases inserted into the genome downstream of the intended edit. Those two cell lines and a third had an observed copy number variant. One clone that appeared to have a normal genome required a greater amount of sorting and passaging to achieve a purified population profile, and had a tendency to differentiate. Since the example presented here contains data for just 3 replicate transfections, it is a too small a dataset to reliably indicate relationships between experimental details and cell line characteristics. However, it is clear that the level of detail of information that can be tracked facilitates comparison of clones with respect to characteristics such as numbers of passages, numbers of days between passaging, phenotypic properties, genomic abnormalities, etc. With a larger dataset, one could evaluate the predictability of the number of clones derived from a transfection protocol, and compare the effect of protocol changes, such as guide RNA, or parent cell line, on transfection efficiency. One could also assess, for example, whether larger numbers of passages or a higher rate of genomic editing in the population is associated with a greater frequency of genomic abnormalities.

## Conclusions

The approach described here enables the collection and sharing of protocol details, metadata and data generated by experimentalists. This project was designed to track protocol details, data, and outcomes during the creation of cell lines by genome engineering. Our intention for this report is not to provide a turnkey software product, but to provide a starting point for other experimental applications that aim to enhance predictability and repeatability in experimental systems that involve many manual and sequential activities with multiple samples. We encourage any laboratory to create a data capture system using software that many biologists are familiar with and customize it for their specific experiments, workflow, and variables. Many features of spreadsheet programs such as the one used here are easily adaptable without deep programming knowledge. Simple reports, and filtering and sorting of data, are easy to perform with built-in functions within the software as shown by this example. While the example presented here is a too small a dataset to reliably indicate relationships between experimental details and cell line characteristics, the electronic collection and organization of many details during complex laboratory processes provide the opportunity to query larger datasets for potential causal relationships between protocols and outcomes.

The need for flexibility and easy customization without requiring modification of underlying code has inspired a recent effort called OMeta^10^, which is an activity designed to address the collection of detailed metadata within the context of the experiment. The OMeta approach is event-based, similar to our approach based on Activities. Two considerations were primary drivers of our approach. One was to design a framework for collecting metadata that was based on, and compatible with, a desired technical workflow, and provide ease of use and benefit to the experimentalist during laboratory activities. A second important consideration was that parameter space should be readily adaptable and expandable by the experimentalist as needed in order to take into account unexpected changes in protocols or the recognition of newly appreciated variables that need to be tracked.

The spreadsheet program used here is useful tool for achieving these goals for the following reasons: it is software that many biologists are familiar with, it is intuitive to use, it is highly flexible and adaptable to specific applications, it has useful and sophisticated built-in functions that are accessible from the toolbar, and daily data entry was easily made part of a normal workflow. In addition, simple queries can be performed by sorting and filtering functions, and the collection of input data in a single table facilitates exporting of the data to a database.

There are potential disadvantages of using a spreadsheet program. Some desirable features found in dedicated programming and database tools such as version control and multiple simultaneous users can be enabled in Excel but with additional effort from the user. Also, the number of lines for entry of data (e.g. activities associated with **Calendar** entries, which for this test case was approximately 1000) is limited to 1,000,000, which could potentially be an issue for very large projects. However, because of the degree of flexibility and ease of use, the spreadsheet format is at least an effective way for the experimentalist to prototype their metadata collection process.

The development of consensus metadata vocabulary has been taking place in various biological specialty areas, for example^11,12^. These activities are critical for establishing consensus on what parameters should be reported for biological experiment data and what terms should be used. Future iterations of our efforts will focus on connecting to standardized metadata where it exists and as it develops.

Making metadata capture and organization as user-friendly as possible and compatible with the experimentalist’s workflow is essential to enable compliance with modern data needs. The strategy presented here could improve reproducibility within a laboratory by making more details available to the next user of the protocols. In addition, such a strategy can make data sharing more effective by providing sufficient details about how the data were collected. This approach provides a mechanism by which the results of a study can be explored in depth by querying the relationship between outcomes and experimental variables. While this strategy can facilitate experimental data tracking locally, it is easily extended to conform to FAIR (Findable, Accessible, Interoperable, Reusable) data principles^13^ and consensus nomenclature and ontologies, and in this way, facilitate data reporting and data sharing.

## Supplementary Information

Supplementary Information.

## Data Availability

The Excel program file, TransfectionTracker1221.xlsm, with its supporting VBA code and a README document, are publicly available at https://github.com/usnistgov/TransfectionTracker.

## References

[1] Chen, S., Alderete, K. A. & Ball, A. RDA Metadata Directory [cited 2022 4/11/2022]. https://rd-alliance.github.io/metadata-directory/subjects/cell-biology.html.

[2] Bucher E, Claunch CJ, Hee D, Smith RL, Devlin K, Thompson W (2019). Annot: a Django-based sample, reagent, and experiment metadata tracking system. BMC Bioinform..

[3] Kanza S, Willoughby C, Gibbins N, Whitby R, Frey JG, Erjavec J (2017). Electronic lab notebooks: can they replace paper?. J. Cheminform..

[4] Protocols.io. [cited 2022 4/20/2022]. https://www.protocols.io/welcome.

[5] Kim S, Kim D, Cho SW, Kim J, Kim JS (2014). Highly efficient RNA-guided genome editing in human cells via delivery of purified Cas9 ribonucleoproteins. Genome Res..

[6] Koch B, Nijmeijer B, Kueblbeck M, Cai Y, Walther N, Ellenberg J (2018). Generation and validation of homozygous fluorescent knock-in cells using CRISPR-Cas9 genome editing. Nat Protoc..

[7] Paquet D, Kwart D, Chen A, Sproul A, Jacob S, Teo S (2016). Efficient introduction of specific homozygous and heterozygous mutations using CRISPR/Cas9. Nature.

[8] Roberts B, Haupt A, Tucker A, Grancharova T, Arakaki J, Fuqua MA (2017). Systematic gene tagging using CRISPR/Cas9 in human stem cells to illuminate cell organization. Mol. Biol. Cell..

[9] Zuris JA, Thompson DB, Shu Y, Guilinger JP, Bessen JL, Hu JH (2015). Cationic lipid-mediated delivery of proteins enables efficient protein-based genome editing in vitro and in vivo. Nat. Biotechnol..

[10] Singh I, Kuscuoglu M, Harkins DM, Sutton G, Fouts DE, Nelson KE (2019). OMeta: an ontology-based, data-driven metadata tracking system. BMC Bioinform..

[11] BioPortal. [cited 2022 4/20/2022]. https://bioportal.bioontology.org/.

[12] Open Microscopy Environment. [cited 2021 4/28/2021]. https://www.openmicroscopy.org/bio-formats/.

[13] Wilkinson MD, Dumontier M, Aalbersberg IJ, Appleton G, Axton M, Baak A (2016). The FAIR Guiding Principles for scientific data management and stewardship. Sci Data..

