# Supplementary material for: Contemporaneous sample data tracking for the generation of genome edited cell lines
Source: Sci Rep. 2022 Dec 9;12:21359. doi: 10.1038/s41598-022-24928-6 (PMC9734375; doi:10.1038/s41598-022-24928-6)
Supplement: Supplementary file 1 — Supplementary Information. [file 41598_2022_24928_MOESM1_ESM.pdf]

Supplemental Information.

The information included here is in the form of screenshots or tables from data in TransfectionTracker1221.xlsm that can be found at https://github.com/usnistgov/TransfectionTracker

In this document, **Bold** lettering indicates the name of a worksheet. ***Bold Italics*** indicates an input available on a worksheet such as ***Activity*** and ***Cell Sample Name*** on the **Calendar** or ***Data*** from the toolbar at the top of the page. *Italics* indicates a choice of input often from a drop-down list. Underline indicates examples of free text.

Figure S1, related to Section 3.1, Data entry worksheets. The relationship between the Cell Samples and Calendar worksheets. Left. The **Cell Samples** worksheet (partial view from a screen shot). The worksheet is automatically updated with cell sample names when the ***Activity*** *Transfect* is chosen on the **Calendar** initiating the creation of a new cell sample, and as derivative cell samples are created by performing activities on that sample. See Table 3 of the manuscript for the list of activities that can be selected from the ***Activity*** drop-down menu on the calendar. The initial cell sample designator is appended with qualifiers (such as *_Sort#*, and *_C6*), which distinguish that sample as unique, but connect it clearly to the original transfected sample. **Right.** A drop-down menu in the **Calendar** worksheet for selection of a cell sample name (partial view). This drop-down menu is automatically populated by the continuously updated **Cell Samples** worksheet. The **Calendar** reflects the dates the action was taken; the ***Date Created*** column in the **Cell Samples** worksheet reflects the actual date the data were entered into the **Calendar**; due to program development challenges, some data had to be reentered in the **Calendar** on a date after the actual date that the activity occurred, and this is reflected in the column, ***Date Created.***

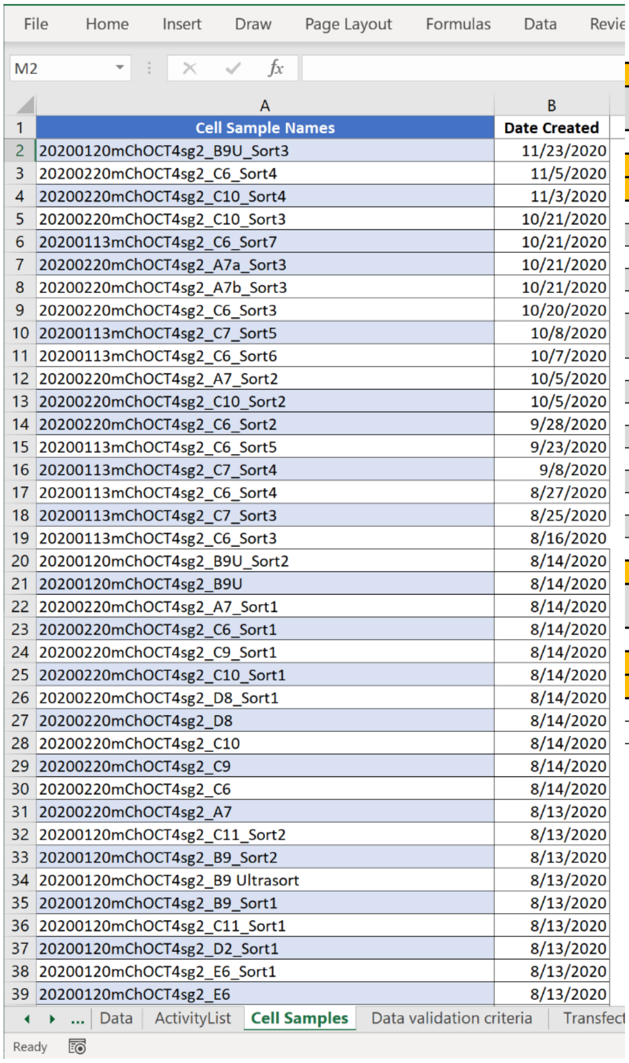

	A	B
1	Cell Sample Names	Date Created
2	20200120mChOCT4sg2_B9U_Sort3	11/23/2020
3	20200220mChOCT4sg2_C6_Sort4	11/5/2020
4	20200220mChOCT4sg2_C10_Sort4	11/3/2020
5	20200220mChOCT4sg2_C10_Sort3	10/21/2020
6	20200113mChOCT4sg2_C6_Sort7	10/21/2020
7	20200220mChOCT4sg2_A7a_Sort3	10/21/2020
8	20200220mChOCT4sg2_A7b_Sort3	10/21/2020
9	20200220mChOCT4sg2_C6_Sort3	10/20/2020
10	20200113mChOCT4sg2_C7_Sort5	10/8/2020
11	20200113mChOCT4sg2_C6_Sort6	10/7/2020
12	20200220mChOCT4sg2_A7_Sort2	10/5/2020
13	20200220mChOCT4sg2_C10_Sort2	10/5/2020
14	20200220mChOCT4sg2_C6_Sort2	9/28/2020
15	20200113mChOCT4sg2_C6_Sort5	9/23/2020
16	20200113mChOCT4sg2_C7_Sort4	9/8/2020
17	20200113mChOCT4sg2_C6_Sort4	8/27/2020
18	20200113mChOCT4sg2_C7_Sort3	8/25/2020
19	20200113mChOCT4sg2_C6_Sort3	8/16/2020
20	20200120mChOCT4sg2_B9U_Sort2	8/14/2020
21	20200120mChOCT4sg2_B9U	8/14/2020
22	20200220mChOCT4sg2_A7_Sort1	8/14/2020
23	20200220mChOCT4sg2_C6_Sort1	8/14/2020
24	20200220mChOCT4sg2_C9_Sort1	8/14/2020
25	20200220mChOCT4sg2_C10_Sort1	8/14/2020
26	20200220mChOCT4sg2_D8_Sort1	8/14/2020
27	20200220mChOCT4sg2_D8	8/14/2020
28	20200220mChOCT4sg2_C10	8/14/2020
29	20200220mChOCT4sg2_C9	8/14/2020
30	20200220mChOCT4sg2_C6	8/14/2020
31	20200220mChOCT4sg2_A7	8/13/2020
32	20200120mChOCT4sg2_C11_Sort2	8/13/2020
33	20200120mChOCT4sg2_B9_Sort2	8/13/2020
34	20200120mChOCT4sg2_B9 Ultrasort	8/13/2020
35	20200120mChOCT4sg2_B9_Sort1	8/13/2020
36	20200120mChOCT4sg2_C11_Sort1	8/13/2020
37	20200120mChOCT4sg2_D2_Sort1	8/13/2020
38	20200120mChOCT4sg2_E6_Sort1	8/13/2020
39	20200120mChOCT4sg2_E6	8/13/2020

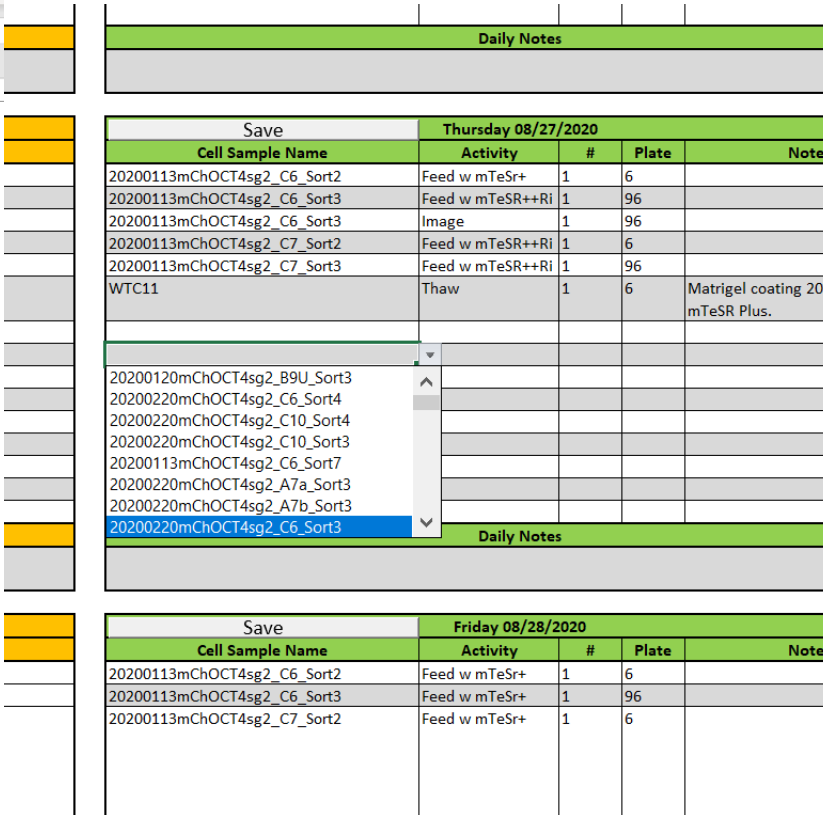

Daily Notes

Save	Thursday 08/27/2020			
Cell Sample Name	Activity	#	Plate	Note
20200113mChOCT4sg2_C6_Sort2	Feed w mTeSr+	1	6	
20200113mChOCT4sg2_C6_Sort3	Feed w mTeSR++Ri	1	96	
20200113mChOCT4sg2_C6_Sort3	Image	1	96	
20200113mChOCT4sg2_C7_Sort2	Feed w mTeSR++Ri	1	6	
20200113mChOCT4sg2_C7_Sort3	Feed w mTeSR++Ri	1	96	
WTC11	Thaw	1	6	Matrigel coating 20 mTeSR Plus.

Drop-down list:
20200120mChOCT4sg2_B9U_Sort3
20200220mChOCT4sg2_C6_Sort4
20200220mChOCT4sg2_C10_Sort4
20200220mChOCT4sg2_C10_Sort3
20200113mChOCT4sg2_C6_Sort7
20200220mChOCT4sg2_A7a_Sort3
20200220mChOCT4sg2_A7b_Sort3
20200220mChOCT4sg2_C6_Sort3

Daily Notes

Save	Friday 08/28/2020			
Cell Sample Name	Activity	#	Plate	Note
20200113mChOCT4sg2_C6_Sort2	Feed w mTeSr+	1	6	
20200113mChOCT4sg2_C6_Sort3	Feed w mTeSr+	1	96	
20200113mChOCT4sg2_C7_Sort2	Feed w mTeSr+	1	6	

Figure S2, related to Section 3.2, Restricted data entry. The Data validation worksheet. *This toolbar-based function allows an experimentalist to easily create lists that limit the selection of variables that can be chosen in the other worksheets.*

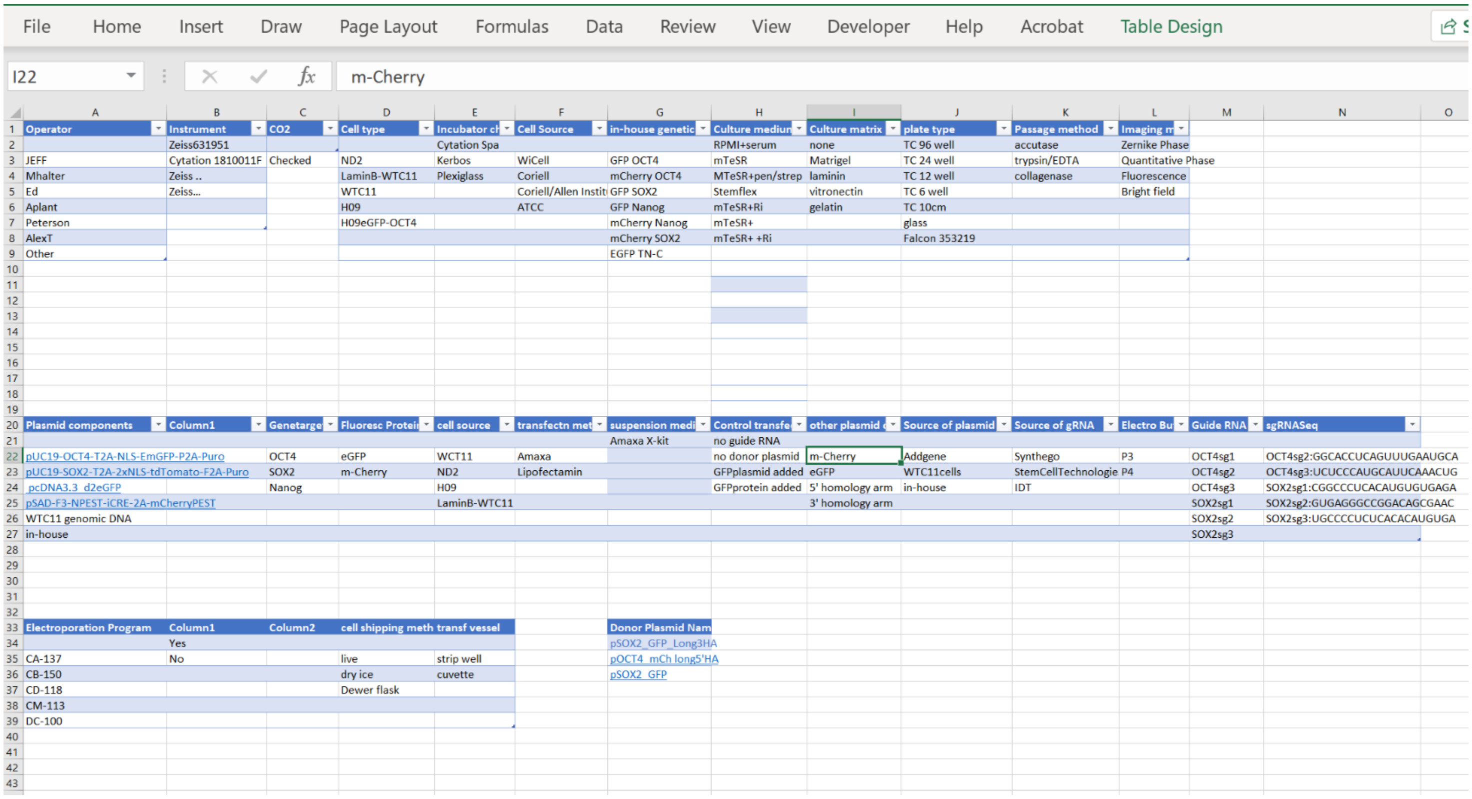

	A	B	C	D	E	F	G	H	I	J	K	L
1	Operator	Instrument	CO2	Cell type	Incubator ch	Cell Source	in-house genetic	Culture medium	Culture matrix	plate type	Passage method	Imaging m
2		Zeiss631951			Cytation Spa			RPMI+serum	none	TC 96 well	accutase	Zernike Phase
3	JEFF	Cytation 1810011F	Checked	ND2	Kerbos	WiCell	GFP OCT4	mTeSR	Matrigel	TC 24 well	trypsin/EDTA	Quantitative Phase
4	Mhalter	Zeiss ..		LaminB-WTC11	Plexiglass	Coriell	mCherry OCT4	MTeSR+pen/strep	laminin	TC 12 well	collagenase	Fluorescence
5	Ed	Zeiss...		WTC11		Coriell/Allen Instit	GFP SOX2	Stemflex	vitronectin	TC 6 well		Bright field
6	Aplant			H09		ATCC	GFP Nanog	mTeSR+Ri	gelatin	TC 10cm		
7	Peterson			H09eGFP-OCT4			mCherry Nanog	mTeSR+		glass		
8	AlexT						mCherry SOX2	mTeSR+ +Ri		Falcon 353219		
9	Other						EGFP TN-C					

	A	B	C	D	E	F	G	H	I	J	K	L	M	N
20	Plasmid components	Column1	Genetarge	Fluoresc Protei	cell source	transfectn met	suspension medi	Control transfe	other plasmid c	Source of plasmid	Source of gRNA	Electro Bu	Guide RNA	sgRNASeq
21							Amaxa X-kit	no guide RNA						
22	pUC19-OCT4-T2A-NLS-EmGFP-P2A-Puro		OCT4	eGFP	WCT11	Amaxa		no donor plasmid	m-Cherry	Addgene	Synthego	P3	OCT4sg1	OCT4sg2:GGCACCUCAGUUUGAAUGCA
23	pUC19-SOX2-T2A-2xNLS-tdTomato-F2A-Puro		SOX2	m-Cherry	ND2	Lipofectamin		GFPplasmid added	eGFP	WTC11cells	StemCellTechnologie	P4	OCT4sg2	OCT4sg3:UCUCCCAUGCAUUCAAACUG
24	pcDNA3.3_d2eGFP		Nanog		H09			GFPprotein added	5' homology arm	in-house	IDT		OCT4sg3	SOX2sg1:CGGCCCUCACAUGUGUGAGA
25	pSAD-F3-NPEST-iCRE-2A-mCherryPEST				LaminB-WTC11				3' homology arm				SOX2sg1	SOX2sg2:GUGAGGGCCGGACAGCGAAC
26	WTC11 genomic DNA												SOX2sg2	SOX2sg3:UGCCCCUCUCACACAUGUGA
27	in-house												SOX2sg3	

	A	B	C	D	E	F	G
33	Electroporation Program	Column1	Column2	cell shipping meth	transf vessel		Donor Plasmid Nam
34		Yes					pSOX2_GFP_Long3HA
35	CA-137	No		live	strip well		pOCT4_mCh long5'HA
36	CB-150			dry ice	cuvette		pSOX2_GFP
37	CD-118			Dewer flask			
38	CM-113						
39	DC-100						

Figure S3, related to Section 3.3, Worksheets for organizing and comparing data. The Data worksheet *(a partial view from a screen shot).* This worksheet is formed from all data, for all samples and all activities, that are entered in the **Calendar** worksheet. The data in this table can be easily exported as an XML or CSV file, or converted into JSON.

	Date	Row	Cell Sample Name	Activity	#	Plate	Notes
403	9/15/2020	7	20200113mChOCT4sg2_C6_Sort4	Feed w mTeSr+	1	96	
404	9/15/2020	9	20200113mChOCT4sg2_C7_Sort2	Feed w mTeSr+	1	6	
405	9/15/2020	8	20200113mChOCT4sg2_C7_Sort3	Feed w mTeSr+	1	12	
406	9/15/2020	10	WTC11	Thaw	1	6	Thawed in mTeSR+Ri.
407	9/16/2020	7	20200113mChOCT4sg2_C6_Sort4	Image	1	96	Used mTeSR w/o phenol red. Then returned to mTeSR Plus.
408	9/16/2020	9	20200113mChOCT4sg2_C7_Sort2	Passage	1	6	Used ReLSR for passage. Transferred 250 of 750 uL into new well of 6 well.
409	9/16/2020	8	20200113mChOCT4sg2_C7_Sort3	Passage	1	6	
410	9/16/2020	10	WTC11	Feed w Ri	1	6	
411	9/18/2020	7	20200113mChOCT4sg2_C6_Sort4	Passage	1	12	Used Accutase into mTeSRPlus+Ri
412	9/18/2020	9	20200113mChOCT4sg2_C7_Sort2	Passage	1	6	Used ReLSR into mTeSRPlus+Ri
413	9/18/2020	8	20200113mChOCT4sg2_C7_Sort3	Passage	5	6	Used ReLSR into mTeSRPlus+Ri
414	9/18/2020	10	WTC11	Passage	1	6	Used ReLSR into mTeSR+Ri
415	9/20/2020	8	20200113mChOCT4sg2_C6_Sort4	Feed w mTeSr+	1	12	
416	9/20/2020	10	20200113mChOCT4sg2_C7_Sort2	Feed w mTeSr+	1	6	
417	9/20/2020	9	20200113mChOCT4sg2_C7_Sort3	Feed w mTeSr+	5	6	
418	9/20/2020	7	WTC11		1	6	
419	9/21/2020	8	20200113mChOCT4sg2_C6_Sort4	Feed w mTeSr+	1	12	
420	9/21/2020	10	20200113mChOCT4sg2_C7_Sort2	Feed w mTeSr+	1	6	
421	9/21/2020	9	20200113mChOCT4sg2_C7_Sort3	Feed w mTeSr+	5	6	
422	9/21/2020	7	WTC11		1	6	
423	9/23/2020	7	20200113mChOCT4sg2_C6_Sort4	Passage	1	6	matrigel-202000903
424	9/23/2020	9	20200113mChOCT4sg2_C7_Sort3	Sort	1	96	Matrigel 20200923. Accutase 5.5 min. Purity sort @<1.3%. Approx 2.4x10e6 total ce min sorted 12778 cells
425	9/23/2020	10	20200113mChOCT4sg2_C7_Sort3	Freeze	5	vials	5 VIALS
426	9/23/2020	8	WTC11	Passage	1	6	matrigel-20200903
427	9/24/2020	8	20200113mChOCT4sg2_C6_Sort4	Feed w mTeSR++Ri	5	6	
428	9/24/2020	9	20200113mChOCT4sg2_C7_Sort3	Feed w mTeSR++Ri	1	96	
429	9/24/2020	7	WTC11	Feed	1	6	
430	9/25/2020	8	20200113mChOCT4sg2_C6_Sort4	Feed w mTeSr+	5	6	
431	9/25/2020	9	20200113mChOCT4sg2_C7_Sort4	Feed w mTeSR++Ri	1	96	
432	9/25/2020	7	WTC11		1	6	mTeSR
433	9/27/2020	8	20200113mChOCT4sg2_C6_Sort4	Feed w mTeSr+	5	6	
434	9/27/2020	9	20200113mChOCT4sg2_C7_Sort4	Feed w mTeSr+	1	96	
435	9/27/2020	7	WTC11		1	6	mTeSR
436	9/28/2020	7	20200113mChOCT4sg2_C6_Sort4	Sort	1	96	Matrigel 20200923. Accutase 5.5 min. No 488 laser. Used 561 laser for F/S scatter ar sorting. WTC11 used for gating out non-mCherry cell population. Ran ~1x10e6 cells collected 27,141 cells in 3.75 min. using UltraPurity setting @ ~35% gate.
437	9/28/2020	8	20200113mChOCT4sg2_C6_Sort4	Feed w mTeSr+	4	6	
438	9/28/2020	9	WTC11	Passage	1	6	mTeSR. Used cells for no mCherry control gating during 20200113mChOCT4_c6_S5
439	9/29/2020	8	20200113mChOCT4sg2_C6_Sort4	Freeze	5	vials	5 VIALS
440	9/29/2020	7	20200113mChOCT4sg2_C6_Sort5	Feed w mTeSR++Ri	1	96	
441	9/29/2020	10	20200113mChOCT4sg2_C7_Sort4	Passage	1	12	
442	9/29/2020	9	WTC11		1	6	
443	9/30/2020	7	20200113mChOCT4sg2_C6_Sort5	Passage	1	12	
444	9/30/2020	8	20200113mChOCT4sg2_C7_Sort4	Feed w mTeSR++Ri	1	12	
445	9/30/2020	9	20200220mChOCT4sg2_A7_Sort1	Thaw	1	6	Thaw in mTeSR+Ri
446	9/30/2020	11	20200220mChOCT4sg2_C10_Sort1	Thaw	1	6	Thaw in mTeSR+Ri

Figure S4, related to Section 3.3, Worksheets for organizing and comparing data. Report Worksheets. Left, a screenshot of the **FreezeReport** worksheet which includes data for all samples from all transfections that were subjected to the ***Activity** Freeze*. **Right,** a partial view of the report worksheet containing all data for samples resulting from transfection 20200113mChOCT4sg2.

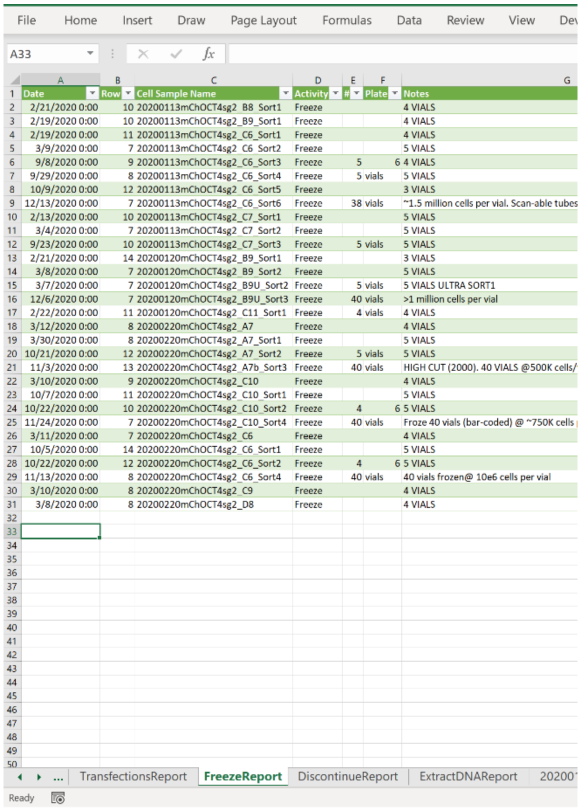

	Date	Row	Cell Sample Name	Activity	#	Plate	Notes
2	2/21/2020 0:00	10	20200113mChOCT4sg2_B8_Sort1	Freeze			4 VIALS
3	2/19/2020 0:00	10	20200113mChOCT4sg2_B9_Sort1	Freeze			4 VIALS
4	2/19/2020 0:00	11	20200113mChOCT4sg2_C6_Sort1	Freeze			4 VIALS
5	3/9/2020 0:00	7	20200113mChOCT4sg2_C6_Sort2	Freeze			5 VIALS
6	9/8/2020 0:00	9	20200113mChOCT4sg2_C6_Sort3	Freeze	5	6	4 VIALS
7	9/29/2020 0:00	8	20200113mChOCT4sg2_C6_Sort4	Freeze	5	vials	5 VIALS
8	10/9/2020 0:00	12	20200113mChOCT4sg2_C6_Sort5	Freeze			3 VIALS
9	12/13/2020 0:00	7	20200113mChOCT4sg2_C6_Sort6	Freeze	38	vials	~1.5 million cells per vial. Scan-able tubes
10	2/13/2020 0:00	10	20200113mChOCT4sg2_C7_Sort1	Freeze			5 VIALS
11	3/4/2020 0:00	7	20200113mChOCT4sg2_C7_Sort2	Freeze			5 VIALS
12	9/23/2020 0:00	10	20200113mChOCT4sg2_C7_Sort3	Freeze	5	vials	5 VIALS
13	2/21/2020 0:00	14	20200120mChOCT4sg2_B9_Sort1	Freeze			3 VIALS
14	3/8/2020 0:00	7	20200120mChOCT4sg2_B9_Sort2	Freeze			5 VIALS
15	3/7/2020 0:00	7	20200120mChOCT4sg2_B9U_Sort2	Freeze	5	vials	5 VIALS ULTRA SORT1
16	12/6/2020 0:00	7	20200120mChOCT4sg2_B9U_Sort3	Freeze	40	vials	>1 million cells per vial
17	2/22/2020 0:00	11	20200120mChOCT4sg2_C11_Sort1	Freeze	4	vials	4 VIALS
18	3/12/2020 0:00	8	20200220mChOCT4sg2_A7	Freeze			4 VIALS
19	3/30/2020 0:00	8	20200220mChOCT4sg2_A7_Sort1	Freeze			5 VIALS
20	10/21/2020 0:00	12	20200220mChOCT4sg2_A7_Sort2	Freeze	5	vials	5 VIALS
21	11/3/2020 0:00	13	20200220mChOCT4sg2_A7b_Sort3	Freeze	40	vials	HIGH CUT (2000). 40 VIALS @500K cells/
22	3/10/2020 0:00	9	20200220mChOCT4sg2_C10	Freeze			4 VIALS
23	10/7/2020 0:00	11	20200220mChOCT4sg2_C10_Sort1	Freeze			5 VIALS
24	10/22/2020 0:00	10	20200220mChOCT4sg2_C10_Sort2	Freeze	4	6	5 VIALS
25	11/24/2020 0:00	7	20200220mChOCT4sg2_C10_Sort4	Freeze	40	vials	Froze 40 vials (bar-coded) @ ~750K cells
26	3/11/2020 0:00	7	20200220mChOCT4sg2_C6	Freeze			4 VIALS
27	10/5/2020 0:00	14	20200220mChOCT4sg2_C6_Sort1	Freeze			5 VIALS
28	10/22/2020 0:00	12	20200220mChOCT4sg2_C6_Sort2	Freeze	4	6	5 VIALS
29	11/13/2020 0:00	8	20200220mChOCT4sg2_C6_Sort4	Freeze	40	vials	40 vials frozen@ 10e6 cells per vial
30	3/10/2020 0:00	8	20200220mChOCT4sg2_C9	Freeze			4 VIALS
31	3/8/2020 0:00	8	20200220mChOCT4sg2_D8	Freeze			4 VIALS

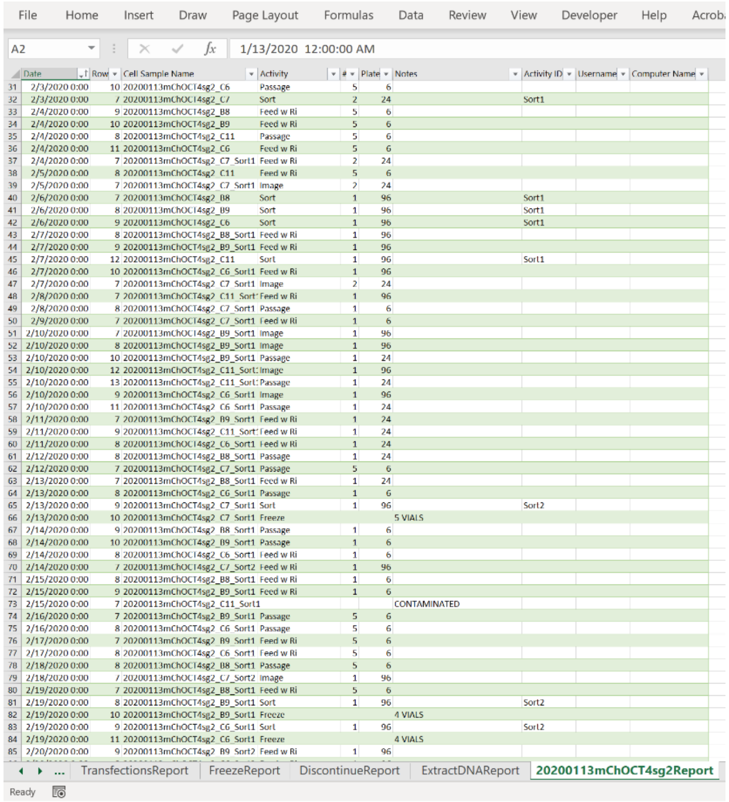

	Date	Row	Cell Sample Name	Activity	#	Plate	Notes	Activity ID	Username	Computer Name
31	2/3/2020 0:00	10	20200113mChOCT4sg2_C6	Passage	5	6				
32	2/3/2020 0:00	7	20200113mChOCT4sg2_C7	Sort	2	24		Sort1		
33	2/4/2020 0:00	9	20200113mChOCT4sg2_B8	Feed w Ri	5	6				
34	2/4/2020 0:00	10	20200113mChOCT4sg2_B9	Feed w Ri	5	6				
35	2/4/2020 0:00	8	20200113mChOCT4sg2_C11	Passage	5	6				
36	2/4/2020 0:00	11	20200113mChOCT4sg2_C6	Feed w Ri	5	6				
37	2/4/2020 0:00	7	20200113mChOCT4sg2_C7_Sort1	Feed w Ri	2	24				
38	2/5/2020 0:00	8	20200113mChOCT4sg2_C11	Feed w Ri	5	6				
39	2/5/2020 0:00	7	20200113mChOCT4sg2_C7_Sort1	Image	2	24				
40	2/6/2020 0:00	7	20200113mChOCT4sg2_B8	Sort	1	96		Sort1		
41	2/6/2020 0:00	8	20200113mChOCT4sg2_B9	Sort	1	96		Sort1		
42	2/6/2020 0:00	9	20200113mChOCT4sg2_C6	Sort	1	96		Sort1		
43	2/7/2020 0:00	8	20200113mChOCT4sg2_B8_Sort1	Feed w Ri	1	96				
44	2/7/2020 0:00	9	20200113mChOCT4sg2_B9_Sort1	Feed w Ri	1	96				
45	2/7/2020 0:00	12	20200113mChOCT4sg2_C11	Sort	1	96		Sort1		
46	2/7/2020 0:00	10	20200113mChOCT4sg2_C6_Sort1	Feed w Ri	1	96				
47	2/7/2020 0:00	7	20200113mChOCT4sg2_C7_Sort1	Image	2	24				
48	2/8/2020 0:00	7	20200113mChOCT4sg2_C11_Sort	Feed w Ri	1	96				
49	2/8/2020 0:00	8	20200113mChOCT4sg2_C7_Sort1	Passage	1	6				
50	2/9/2020 0:00	7	20200113mChOCT4sg2_C7_Sort1	Feed w Ri	1	6				
51	2/10/2020 0:00	7	20200113mChOCT4sg2_B9_Sort1	Image	1	96				
52	2/10/2020 0:00	8	20200113mChOCT4sg2_B9_Sort1	Image	1	96				
53	2/10/2020 0:00	10	20200113mChOCT4sg2_B9_Sort1	Passage	1	24				
54	2/10/2020 0:00	12	20200113mChOCT4sg2_C11_Sort	Image	1	96				
55	2/10/2020 0:00	13	20200113mChOCT4sg2_C11_Sort	Passage	1	24				
56	2/10/2020 0:00	9	20200113mChOCT4sg2_C6_Sort1	Image	1	96				
57	2/10/2020 0:00	11	20200113mChOCT4sg2_C6_Sort1	Passage	1	24				
58	2/11/2020 0:00	7	20200113mChOCT4sg2_B9_Sort1	Feed w Ri	1	24				
59	2/11/2020 0:00	9	20200113mChOCT4sg2_C11_Sort	Feed w Ri	1	24				
60	2/11/2020 0:00	8	20200113mChOCT4sg2_C6_Sort1	Feed w Ri	1	24				
61	2/12/2020 0:00	8	20200113mChOCT4sg2_B8_Sort1	Passage	1	24				
62	2/12/2020 0:00	7	20200113mChOCT4sg2_C7_Sort1	Passage	5	6				
63	2/13/2020 0:00	7	20200113mChOCT4sg2_B8_Sort1	Feed w Ri	1	24				
64	2/13/2020 0:00	8	20200113mChOCT4sg2_C6_Sort1	Passage	1	6				
65	2/13/2020 0:00	9	20200113mChOCT4sg2_C7_Sort1	Sort	1	96		Sort2		
66	2/13/2020 0:00	10	20200113mChOCT4sg2_C7_Sort1	Freeze			5 VIALS			
67	2/14/2020 0:00	9	20200113mChOCT4sg2_B8_Sort1	Passage	1	6				
68	2/14/2020 0:00	10	20200113mChOCT4sg2_B9_Sort1	Passage	1	6				
69	2/14/2020 0:00	8	20200113mChOCT4sg2_C6_Sort1	Feed w Ri	1	6				
70	2/14/2020 0:00	7	20200113mChOCT4sg2_C7_Sort2	Feed w Ri	1	96				
71	2/15/2020 0:00	8	20200113mChOCT4sg2_B8_Sort1	Feed w Ri	1	6				
72	2/15/2020 0:00	9	20200113mChOCT4sg2_B9_Sort1	Feed w Ri	1	6				
73	2/15/2020 0:00	7	20200113mChOCT4sg2_C11_Sort1				CONTAMINATED			
74	2/16/2020 0:00	7	20200113mChOCT4sg2_B9_Sort1	Passage	5	6				
75	2/16/2020 0:00	8	20200113mChOCT4sg2_C6_Sort1	Passage	5	6				
76	2/17/2020 0:00	7	20200113mChOCT4sg2_B9_Sort1	Feed w Ri	5	6				
77	2/17/2020 0:00	8	20200113mChOCT4sg2_C6_Sort1	Feed w Ri	5	6				
78	2/18/2020 0:00	8	20200113mChOCT4sg2_B8_Sort1	Passage	5	6				
79	2/18/2020 0:00	7	20200113mChOCT4sg2_C7_Sort2	Image	1	96				
80	2/19/2020 0:00	7	20200113mChOCT4sg2_B8_Sort1	Feed w Ri	5	6				
81	2/19/2020 0:00	8	20200113mChOCT4sg2_B9_Sort1	Sort	1	96		Sort2		
82	2/19/2020 0:00	10	20200113mChOCT4sg2_B9_Sort1	Freeze			4 VIALS			
83	2/19/2020 0:00	9	20200113mChOCT4sg2_C6_Sort1	Sort	1	96		Sort2		
84	2/19/2020 0:00	11	20200113mChOCT4sg2_C6_Sort1	Freeze			4 VIALS			
85	2/20/2020 0:00	9	20200113mChOCT4sg2_B9_Sort2	Feed w Ri	1	96				

Table S1, related to Section 3.3, Worksheets for organizing and comparing data. Worksheet for calculating passage numbers. Data associated with the cell line 20200220mChOCT4sg2_A7 were copied from the **20200220mChOCT4sg2Report** worksheet and pasted into a new worksheet. Rows of data were selected using the ***Activity*** column to choose records for activities related to advancing passage number. Passaging events can be easily counted and this disambiguates the passage number of frozen samples that are returned to culture with the ***Activity*** *Thaw*, or at which frozen banks of cells are created, or of samples used for genomic analysis. A bifurcation of this sample (and when it occurred) is clear by the modified name _A7b. These data are in the worksheet **Passage#s20200220_A7**.

Date	Cell Sample Name	Activity	Passage count	#	Plate	# Passages	
2/20/2020 0:00	20200220mChOCT4sg2	Transfect		96	96		
3/1/2020 0:00	20200220mChOCT4sg2_A7	Passage	1	1	24		
3/5/2020 0:00	20200220mChOCT4sg2_A7	Passage	1	1	6		
3/9/2020 0:00	20200220mChOCT4sg2_A7	Passage	1	5	6		
3/12/2020 0:00	20200220mChOCT4sg2_A7	Freeze				3	
3/22/2020 0:00	20200220mChOCT4sg2_A7_Sort1	Passage	1	1	24		
3/25/2020 0:00	20200220mChOCT4sg2_A7_Sort1	Passage	1	1	6		
3/27/2020 0:00	20200220mChOCT4sg2_A7_Sort1	Passage	1	5	6		
3/30/2020 0:00	20200220mChOCT4sg2_A7_Sort1	Freeze				6	
9/30/2020 0:00	20200220mChOCT4sg2_A7_Sort1	Thaw	1	1	6		
10/2/2020 0:00	20200220mChOCT4sg2_A7_Sort1	Passage	1	5	6		
10/12/2020 0:00	20200220mChOCT4sg2_A7_Sort2	Passage	1	1	12		
10/15/2020 0:00	20200220mChOCT4sg2_A7_Sort2	Passage	1	1	6		
10/17/2020 0:00	20200220mChOCT4sg2_A7_Sort2	Passage	1	5	6		
10/21/2020 0:00	20200220mChOCT4sg2_A7_Sort2	Freeze		5		11	
10/27/2020 0:00	20200220mChOCT4sg2_A7_Sort2	Extract DNA				**11**	
10/28/2020 0:00	20200220mChOCT4sg2_A7b_Sort3	Passage	1	6	6		
10/31/2020 0:00	20200220mChOCT4sg2_A7b_Sort3	Passage	1	1	6		
10/31/2020 0:00	20200220mChOCT4sg2_A7b_Sort3	Passage	1	5	10		
11/3/2020 0:00	20200220mChOCT4sg2_A7b_Sort3	Freeze		40		14	
11/9/2020 0:00	20200220mChOCT4sg2_A7b_Sort3	Passage	1	1	6		
11/16/2020 0:00	20200220mChOCT4sg2_A7b_Sort3	Passage	1	1	6		16
11/19/2020 0:00	20200220mChOCT4sg2_A7b_Sort3	Thaw	1	1	6	15	
11/22/2020 0:00	20200220mChOCT4sg2_A7b_Sort3	Passage	1	2	6		
11/25/2020 0:00	20200220mChOCT4sg2_A7b_Sort3	Passage	1				
11/29/2020 0:00	20200220mChOCT4sg2_A7b_Sort3	Extract DNA		1		**17**	
12/3/2020 0:00	20200220mChOCT4sg2_A7b_Sort3	Send out for analysis				17	
1/11/2021 0:00	20200220mChOCT4sg2_A7b_Sort3	Thaw				15	
1/13/2021 0:00	20200220mChOCT4sg2_A7b_Sort3	Send out for analysis				**15**	

Table S2, related to Section 3.3, Worksheets for organizing and comparing data. The collated results of the fate of clones. Some of the data in this table were added through automated queries, other data were manually entered. This sheet indicates which clones were analyzed and which were discontinued, the date that analysis was initiated and the passage number of the cell sample at that time, and results such as copy number of the fluorescent protein sequence, copy number variants, results of PCR analysis in the insertion site, qualitative appearance of the cell line, and other analyses such as for mycoplasma and for STR markers. Because the URL where the data for each clone is saved appears in the table, it is easy to identify and access the primary data unambiguously. These data are in the worksheet **ClonesSummaries.**

CloneID	Address	Discontinued Notes	DNA extraction date	Sent for analysis date	Sort#	Passage #	FP Copy #	CopyNumber Variants (Stemgenomics)	In-house Junctional PCR	Rel intens (flow)	qual appearance	Mycoplasma (ATCC)	STR (ATCC)
20200113mChOCT4sg2_B8	\\129.6.187.15\Data\EP	Lost fluorescence											
20200113mChOCT4sg2_B9	\\129.6.187.15\Data\EP	Lost fluorescence											
20200113mChOCT4sg2_C11	\\129.6.187.15\Data\EP	Contamination											
20200113mChOCT4sg2_C6	\\129.6.187.15\Data\EP		11/29/2020	12/3/2020	5	23	1.02	none	inconclusive	most not bright, but maybe a subpop that is bright	tendency to differentiate?		
			12/14/2021	12/18/2021	6	26	1.06	none					
				1/13/2021	6	28						Negative	Match to WTC11
20200113mChOCT4sg2_C7	\\129.6.187.15\Data\EP	fluorescence unstable?											
20200120mChOCT4sg2_B9U	\\129.6.187.15\Data\EP		12/14/2020		3	18	1.08	Chr20q (3)	300bp insertion downstream	bright			
				1/13/2021	3	20						Negative	Match to WTC11
20200120mChOCT4sg2_C11	\\129.6.187.15\Data\EP	Lost fluorescence											
20200120mChOCT4sg2_D2	\\129.6.187.15\Data\EP	LOST-passage too dilute cells never grew out.											
20200120mChOCT4sg2_E6	\\129.6.187.15\Data\EP	LOST-passage too dilute cells never grew out.											
20200220mChOCT4sg2_A7b	\\129.6.187.15\Data\EP		11/29/2020		3	17	0.96	Chr11 suspect	3Kbp insertion downstream	bright			
				1/13/2021		15						Negative	Match to WTC11
20200220mChOCT4sg2_C10	\\129.6.187.15\Data\EPCL\20200220mC		11/29/2020	1/13/2021	4	15	1.14	Chr11 suspect		not bright	tendency to differentiate?	Negative	Match to WTC11
20200220mChOCT4sg2_C6	\\129.6.187.15\Data\EPCL\20200220mC		11/29/2020	1/13/2021	4	17	0.95	none		bright		Negative	Lost allele in PentaE locus
20200220mChOCT4sg2_C9	\\129.6.187.15\Data\EP	Lost fluorescence											
20200220mChOCT4sg2_D8	\\129.6.187.15\Data\EP	Lost fluorescence and strange blob morphology appeared.											

Note S1, related to Section 2.3, System architecture design. This README file is found at https://github.com/usnistgov/TransfectionTracker

The following is the full content of that file and includes instructions for how to use TransfectionTracker and how to modify the existing program to adapt it to a different use-case.

TransfectionTracker README

Disclaimer: This program is distributed in the hope that it will be useful, but WITHOUT ANY WARRANTY that it will perform flawlessly or be fit for a particular purpose. The use or mention by NIST of commercial products does not imply endorsement or indication that they are the only, or best, products.

Purpose of this program

This program allows collection of information about gene editing operations and activities performed on transfected cells. It allows tracking of what is done and when to individual cell samples over time.

Instructions for using TransfectionTracker1221

TransfectionTracker1221.xlsm

Table of contents

GENERAL OVERVIEW

DATA ENTRY: STEP-BY-STEP

FEATURES OVERVIEW

1) The **Calendar** worksheet
2) Navigating the **Calendar** worksheet
3) Using the *#* and *Plate* columns
4) ***Activity*** *Discontinue*
5) ***Activity*** *Thaw*
6) ***Activity*** *Sort*
7) Other worksheets
8) Controlling and making changes to the worksheets
9) **Do NOT…**

GENERAL OVERVIEW:

1) This program was created in Microsoft Excel for Microsoft 365 MSO (16.0.13127.21490) 32-bit.
2) The program is composed of interrelated worksheets that are accessed by named tabs that run along the bottom of the screen. Worksheets include **Calendar**, **Metadata Template**, **Cell Samples**, **ActivityList**, **Data Validation Criteria**, and **Documentation**.

3) In this document, **Bold** lettering indicates the name of a worksheet. ***Bold Italics*** indicates an input available on a worksheet such as ***Activity*** and ***Cell Sample Name*** on the **Calendar** or ***Data*** from the toolbar at the top of the page. *Italics* indicates a choice of input often from a drop-down list. Underline indicates examples of free text.
4) Two versions of the program are provided. One is populated with existing data so the user can see examples of what data are collected, how data are tabulated, and examples of reports created by querying the data. The version indicated by ".clean" has no saved data. The user can begin to populate this version with new or test data, or they can modify it to suit their own data needs.
5) Refer to the **Documentation** worksheet within the program for additional specific information.

DATA ENTRY: STEP-BY-STEP:

1) On opening the program, in response to dialog boxes, Enable Editing, Enable Content. If you intend to add data, answer NO if you see a question to open in ReadOnly mode.
2) From the **Calendar** worksheet: Type in a date or use blue arrows on upper left of the **Calendar** to navigate.
    a) From ***Activity*** for that date, choose *Transfect.* Use button at upper right of that day to ***Save***. A ***Metadata Sheet Selection*** box will appear; use the drop-down list to choose *Metadata Template* (subsequently, the new metadata sheet that is about to be created will be a choice option). Click ***Proceed***. A message that the data have been saved will appear; click *OK*. The **Metadata Template** will open. Cell D5 will be prepopulated with the selected date.
        i) Provide the minimum amount of information by making a selection from the drop-down lists in cells D11, G11, and D12. You will see error messages in cells L11 and L12 if the ***Original plasmid backbone information*** (box to the right) contains data that are inconsistent with the information in D11, G11 and D12. An error here will not prevent proceeding. A transfection designator will be created in cell D44 with this information and the worksheet will be renamed.
3) Navigate back to the **Calendar** worksheet. The cell sample name that corresponds to the transfection designator (and which is the name of the new metadata worksheet) now appears on the **Calendar** worksheet in the ***Cell Sample Name*** column corresponding to the ***Activity*** *Transfect*. Press the ***Save*** button again. The new transfection designator now appears in the drop-down list under ***Cell Sample Name***.
4) After entering data on the **Calendar**, press the ***Save*** button before navigating away from the **Calendar** page.

FEATURES OVERVIEW:

1) The **Calendar** worksheet allows entry of activities performed on each sample for any date.
2) In the **Calendar** worksheet, navigate to any date desired using the large BLUE arrows in the upper left of the Calendar window, or use the calendar icon to enter a date. In the box for the desired date, use drop-down arrows under headings ***Cell Sample Name*** and ***Activity*** to show lists of cell samples and activities to select from. The names of all cell samples that

have been created will appear in the drop-down list and in the **Cell Samples** worksheet, which is updated when new samples are created.

a) The ***Activity,*** *Transfect,* provides a template for collecting metadata about a new transfection. The user can choose the **Metadata Template** worksheet or a previously created transfection worksheet to modify. As the data for the new transfection are entered in the worksheet, the name of the worksheet is updated to reflect the date (which is automatically entered when the ***Activity,*** *Transfect,* is initiated from the **Calendar**), and other data that the user inputs about that specific transfection including the gene being modified, the fluorescent protein sequence being used and a designation for the guide RNA. A transfection designator (line 44 on the worksheet) is assigned by concatenating this information and the worksheet is automatically renamed with the transfection designator, which is the new cell sample name. Thus for each transfection, a worksheet named with the date and other information about the transfection is created in the workbook. An electronic folder named with the transfection designator can be automatically created within the operating system by providing the url of a network computer in cell A6 of the Documentation worksheet.**

b) When the new transfection worksheet and the corresponding transfection designator are created, the new cell sample name is added to the **Cell Samples** worksheet and becomes available in the drop-down menu in the **Calendar** worksheet under ***Cell Sample Name.*** The newest addition to the **Cell Samples** worksheet will be at the top of the ***Cell Sample Name*** drop-down list.

c) In the use case shown here, each cell sample can be chosen on any day of the **Calendar** for an activity such as feeding, imaging, passaging, etc. (In this use case, the results of imaging measurements were used to determine whether or not there was a fluorescent transfected clone in the well plate.)

d) When a clone is identified, it is given a new name by choosing the button to the right of the date labeled **Designate New Clone**. The user is presented with a drop-down menu containing existing cell sample names from which they make a selection. This is followed by a free text window to assign a *Clone ID*, which is generally a position on a multi-well plate. The *Clone ID* will be appended to the original cell sample name and the newly created name will be added to the **Cell Samples** worksheet and appear in the ***Cell Sample Name*** drop-down list on the **Calendar**. A new subfolder designated with the name of the new clone can be created in the folder that was named for the transfection designator.**

e) Worksheets can be temporarily hidden with the *Hide/Unhide* option by right clicking on the worksheet tab. This will reduce the number of worksheets visible (but accessible) in the workbook.

3) The columns labeled ***#*** and ***Plate*** can be used to record the # of wells used and the total number of wells in cell culture plate for activities such as *Passage*, *Sort*, and *Freeze*. Exceptions to this rule for entries in the ***Plate*** column:

(1) # of 10cm plates (type in 10cm)
(2) # of vials for freezing (type in the word vial)

(3) # of flasks of size (T125) (type in T125 or other flask designation)

4) Select ***Activity*** *Discontinue* when no versions of that clone are being carried further because of loss of fluorescence or other reason. Add reason to the ***Notes*** column as free text.

5) The ***Activity*** *Thaw* applies only to cells from a bank previously subjected to ***Activity*** *Freeze*. If you take a sample from the bank, indicate ***Activity*** *Thaw*. This might be followed by ***Activity*** *ExtractDNA*.
   a) A *Thaw* event is counted as incrementing passage number.

6) ***Activity*** *Sort* will append the **Cell Samples** worksheet with a new cell sample name to indicate sort number (_Sort#). This new name will appear in the ***Cell Sample Name*** drop-down list on the **Calendar**. This action can also initiate the creation of a new folder with the appended name in the appropriate Transfection folder if that feature is enabled.**
   a) Details of ***Activity*** *Sort* can be captured as free text in adjacent ***Notes*** column.
   b) The sort number automatically increments when a previously sorted sample is sorted again.
   c) The ***Activity*** *Sort* is counted as an increment to passage number.

7) Other worksheets:
   a) Existing cell sample names are listed in worksheet **Cell Samples**.
   All activities are listed in worksheet **Activity List**.
   b) Data collected on the **Calendar** worksheet are organized in tabular form in worksheet **Data**. These data can be in the form of a data range or designated as a table by highlighting all cells and selecting from the toolbar ***Insert*** / *Tables* / *Table*.
   c) Examples of three **Transfection Metadata** worksheets (ex. **20200120mChOCT4sg2**) that were created for clones resulting from three transfections.
   d) **The **Documentation** worksheet contains instructions to direct the automatic creation of folders and files with human readable names on a network drive. By default, the lines of code that enable that function are comment lines.
   e) Summary data can be created by queries. Examples of queries are **TransfectionsReport**, **DiscontinuedReport**, **FreezeReport**, **ExtractDNAReport**, **20200113mChOCT4sg2Report** and **Passage#s…** worksheets. As new data are added to the **Data** worksheet (such as from new entries on the **Calendar**, these **…Reports** worksheets can be updated from the toolbar by *Data* / *Refresh All*.

8) Controlling and making changes to the worksheets: [N.B.: These tips are meant to guide a user, not to serve as an Excel tutorial.]
   a) Whether you are using TransfectionTracker1221.xlsm or TransfectionTracker1221.clean.xlsm, consider saving a renamed version that you can write over without changing the original file.
   b) The **Metadata Template** has been designed for a particular use case, i.e., the collecting of information about clonal cell lines that are transfected by electroporation and identified and isolated by their fluorescence signal. The **Metadata Template** is created to capture some of the variations on a general protocol that are likely to occur and/or

are being specifically tested. The metadata capture approach is designed to make it easy to record relevant specific details about the transfection and clonal isolation. Other use cases could require a modification or redesign of the template. This is relatively easy to do even without making changes to the VBA code by leaving in place cells that are specifically referred to in the code, namely D5 (date of transfection) and D44 (the transfection designator). If those positions are maintained, a new template could be created and substituted for the **Metadata Template** presented here. An example of modification of the template is shown by the differences between the metadata worksheet for transfection **20200120mChOCT4sg2** and the **Metadata Template.** The former was modified to add additional options on line 33 of the **Metadata Template** regarding the vessel for the transfection reaction.

i) If a different naming scheme is desired, one can change the concatenation argument in cell D44 of the **Metadata Template**. Unprotect the worksheet as described in 8d below. Cell D44 currently references the values of three cells, D5 (the date), H11 (the fluorescent protein) and D12 (the guide RNA designation). Choose different cells to include in the concatenation instead, or create new cells with the desired new information, and reference those values in D44.

ii) The **Data Validation criteria** worksheet can be edited to add options to existing drop-down lists. With your cursor on the cell that you want to add drop-down options to, choose ***Data*** on the toolbar and the *Data Validation* option under *Data Tools*. In the pop-up box, choose *List*, and as the *Source*, navigate to the **Data Validation** worksheet and highlight the cells that you want the user to be able to select. If you add terms to one of the lists, you will have to expand the size of the list by dragging your cursor to include the added options. You can always eliminate or substitute terms in these lists. If desired, you can protect this sheet to keep anyone else from making changes.

iii) New drop-down lists can be added to the **Data Validation criteria** worksheet and can be selected from another worksheet as described above.

iv) Drop-down lists can be filtered by highlighting the contents, selecting *Data* from the toolbar, and the *Filter* option from *Sort & Filter*.

c) Simple queries (the results of which are shown in the worksheets named **….Report**) can be generated by designating the **Data** worksheet contents as a table, highlighting the data, and selecting from the toolbar ***Data*** / *Get & Transform Data* / *From Table/Range* / *Table* (icon). A *Power Query Editor* dialog box appears, and data of interest can be selected from the ***Activities*** column. A new worksheet with the selected data is created which can be renamed. As new data are added to the **Data** worksheet (such as from new entries on the **Calendar**, these **…Reports** worksheets can be automatically updated from the toolbar by *Data* / *Refresh All*. A similar approach was used to generate the …**Report** worksheets for each transfection by selecting on ***Cell Sample Name*** in the *Power Query Editor* dialog box. Further queries of these selected data were used to generate the **Passage#s…** worksheets. [N.B. Do not assign the **Cell Samples** worksheet as a table, since it needs to operate as a data range.]

d) Protecting and unprotecting workbooks, worksheets, and cells provides control by restricting worksheet cell entries while allowing intentional changes to be made. The

protected status of the workbook and worksheets can be seen by choosing ***File*** / *Info*. Five worksheets in the current workbook are protected: **Calendar**, **Metadata Template** and metadata worksheets for three transfections. Protections can be removed from the ***File*** / *Info* page by pressing *Unprotect*, or from the worksheet at the tool bar by choosing ***Review*** / *Unprotect Sheet*, by using the password aplant. From a worksheet, it is possible to designate, through ***Home*** / *Cells* / *Format* function, which cells are to be protected and which are to be available for user input. In the **Metadata Template** worksheet, the orange-colored cells are not protected so the user can enter or select values for those cells.

e) If entries are removed from the **Data** worksheet, they will be removed from the **Calendar** worksheet as well when the calendar date is scrolled.

f) The activity ***Transfect*** could be renamed to accommodate a different kind of experiment or to use this tracking system for routine handling of multiple cell samples. For example, ***Transfect*** could be substituted in the **ActivityList** for ***Start a new cell line*** and the **Metadata Template** could be modified as described in part 8b above. However, it would be necessary to be sure that the new activity name was substituted in all relevant lines in the VBA code.

9) **Do NOT:**

a) …Type in a cell sample name or an activity into a cell on the **Calendar**. Choose only options from the drop-down lists. If additions to the drop-down lists are needed, add them in the **Data Validation** worksheet.

b) …Insert a row, column or cell into the **Calendar**. If a row is added inadvertently, it may not have a drop-down arrow associated with it. If this happens, simply use a row for that date that does access a drop-down list.
